# Interrelationship of *myo*-inositol pathways with systemic metabolic conditions in two strains of high-performance laying hens during their productive life span

**DOI:** 10.1038/s41598-021-84169-x

**Published:** 2021-02-25

**Authors:** Fernando Gonzalez-Uarquin, Vera Sommerfeld, Markus Rodehutscord, Korinna Huber

**Affiliations:** Institute of Animal Science, University of Hohenheim, 70599 Stuttgart, Germany

**Keywords:** Metabolomics, Predictive markers

## Abstract

Adaptation to metabolic challenges is an individual process in animals and human, most likely based on genetic background. To identify novel pathways of importance for individual adaptation to a metabolic challenge such as egg production in laying hens, *myo*-inositol (MI) metabolism and plasma metabolite profiles during the productive lifespan were examined in two genetically different strains, Lohmann Brown-Classic (LB) and LSL-Classic (LSL) hens. They were housed during the productive lifespan and sampled at 10, 16, 24, 30 and 60 weeks of age. The targeted AbsoluteIDQ p180 Kit was used for metabolite profiling in plasma whereas a MI enzymatic kit and ELISAs were used to quantify tissue MI concentrations and MI key enzymes (IMPase 1 and MIOX), respectively. As major finding, kidney MIOX was differently expressed in LB and LSL hens with higher amounts in LB. The onset of egg laying between week 16 and 24 of life span was associated with a clear change in the metabolite profiles, however LSL hens and LB hens adapt differently. Pearson’s correlation analyses over all hens at all time points indicated that higher expression of MI degrading enzyme MIOX was related to markers indicating metabolic stress.

## Introduction

The lifespan of laying hens is challenged by many metabolic changes to fulfill their production performance. In the post-hatch period, the gastrointestinal tract undergoes rapid morphological and functional developmental changes to adapt from yolk utilisation to solid feed digestion^1^. Around the week 15, reproductive tract started maturation; concomitantly, an increase of fat cells in abdominal fat depots was observed^2^. During this period hens continue growing and maturing, while egg-laying started simultaneously^3^. At about week 24, hens become sexually mature, and egg production increases until reaching its peak around week 30^4^. After week 30, hens growth has been terminated, and egg production gradually decreases, but continues up to 60 weeks of age^5^. Although many high-performance strains are commonly used in egg production industry, it is most likely that different strains cope with metabolic challenges related to egg laying more or less efficient.

Thus, during the last decade attention has been placed on the physiological differences between laying hen strains. Tona et al.^6^ demonstrated that embryos developed faster in Lohmann Brown than in Lohmann White suggesting the incubation conditions should be determined strain-specifically. In adult hens, it was demonstrated that Lohmann Brown showed larger and heavier bones^7^. Brown Hy-line hens presented lower corticosterone concentrations and higher feed intake and egg production while handling stress in comparison to white layers^8^, categorizing brown and white layers as proactive and reactive, respectively^9^. Plasma serotonin concentration, a metabolite associated to energy homeostasis and bird behavior, also was lower in White Leghorn hens compared to Rhode Island Red hens^10^.

It is well known that laying hens required specific nutrition matching the needs of the productive stages^11^. However, knowledge about the metabolic network underlying general and strain-specific adaptation to egg laying is rare. To describe pathways associated with the metabolic phenotypes of many species including chicken, targeted or untargeted metabolomics approaches, based on liquid chromatography–mass spectrometry, have been shown to be powerful instruments^12^. Metabolomics provides a snapshot-like quantification of a certain number of metabolites, which were associated with several metabolic pathways, reflecting a wide range of physiological processes such as energy and amino acid metabolism. Identification of metabolites of interest (MOI), which e.g. differentiate strain-specific adaptive processes, could help generating new hypotheses about novel pathways of metabolic efficiency and health.

Among MOI, *myo*-inositol (MI), a cyclic sugar polyalcohol, was suggested to have a special impact on chicken metabolism, especially in broilers^13,14^. MI is synthesized endogenously from d-glucose or from inositol phosphates dephosphorylation by the action of inositol monophosphatase 1 (IMPase 1); it is catabolized mainly by the action of a non-heme di-iron oxygenase, the *myo*-inositol oxygenase (MIOX) located in the kidney. MI acts as a precursor of phosphatidylinositol (PI), which in turn is part of the glycerophospholipid metabolism involved in cell membrane turn over. Furthermore, MI appeared to be involved in brain osmolarity regulation, energy and lipid metabolism, bone and muscle formation, reproduction, and general metabolic performance^15^. Mainly due to the role of MI in the intracellular inositol trisphosphate (InsP3) metabolism, almost all tissues depend on MI availability. InsP3 and its receptors are involved in regulation of a plethora of metabolic pathways which are modulated by the InsP3-triggered calcium release from intracellular stores and by interactions of the InsP3 receptors with regulatory proteins^16^. Consequently, as one major metabolic effect, MI modulates insulin secretion from pancreatic beta-cells; thereby influencing glucose and lipid metabolism of all insulin-sensitive targets such as liver, muscle and adipose tissues. It was found that liver lipid accumulation could be prevented by MI supplementation in rats fed with high sugar diets^17^. Due to the tight cross-talk of insulin with the adipokine leptin, MI might promote also an increase in energy expenditure and reduction of food intake leading to a decrease in plasma glucose levels in diabetes type 2 patients^18^. Furthermore, MI can be used to intracellularly synthesize inositol hexakisphosphate (phytic acid; InsP6), which is also described to promote various ameliorating effects on metabolism and energy expenditure^19^. A reduction of oxidative stress and an increase in insulin sensitivity are the most striking metabolic outcomes of MI and InsP6 supplementation in human and rats^18^. Improved insulin sensitivity was in part based on an increase in glucose transporter 4 translocation into the plasma membrane of skeletal muscle cells by MI resulting in higher cellular glucose uptake^20^. Consequently, in reproductive physiology, MI is effective in the treatment of polycystic ovary syndrome (PCOS) and metabolic imbalance in women because PCOS is strongly related to insulin resistance^21^. Besides, MI and InsP3 are directly involved in oocyte quality and maturation^21^.

Many of the ameliorating effects of MI cannot be fully explained mechanistically yet; however, there is strong scientific evidence about a profound metabolic importance of MI in human and animal species. For chicken, information is scarce to date^15^. However, insulin insensitivity, metabolic disturbances in lipid and glucose metabolism, poor bone health and infertility are well-known production diseases in high performing laying hens^22,23^. Therefore, a functional MI and InsP6 metabolism might also be important in this species. Although in human studies the impact of dietary InsP6 supplementation on metabolic health was shown, the alimentary approach in animal nutrition is different. Poultry diets are mostly based on grains and their processing by-products and thereby, contain high amounts of phytate. Due to the anti-nutrient properties of InsP6 a complete degradation in the intestine is intended to improve digestibility of minerals (phosphorus, calcium) and other nutrients such as amino acids by adding exogenous microbial phytases to the diet^24^. However, there is scientific evidence that individual chickens are able to be efficient in intestinal phytate degradation by endogenous mucosal phytases and phosphatases to release and to absorb MI^25^. It is hypothesized that the genetic background contributes to defining these special features in regards to MI metabolism. As a consequence, these chickens may have higher plasma MI concentrations associated with signs of metabolic health. Therefore, the aim of this study was to generate novel knowledge about MI and its relationship with energy and amino acid metabolism in two strains of high-performance laying hens along the productive lifespan. It was hypothesized that due to their differences in genotype and phenotype onset of egg-laying was performed differently which may result in more or less efficient metabolic adaptation associated with variations in MI pathways.

## Results and discussion

MI metabolism and all related derivatives from MI-linked pathways—including intestinal release of MI from phytate—are suggested to be crucial for the laying hen^15^. MI availability is determined by absorption from the gut, endogenous synthesis in tissues, and degradation in kidneys. Genetic background and productive period might determine these components of MI metabolism. Thus, this study aimed to describe components of MI metabolism during productive lifespan and to identify individual metabolite profiles which reflect energy and amino acid metabolism, mitochondrial functionality, and chronic inflammation.

### Liver and muscle *myo*-inositol and inositol monophosphatase 1 protein concentrations

Average MI concentrations in the liver of LB and LSL hens were higher at week 60 compared to the other weeks without any effect of strains. This was paralleled by the concentrations of IMPase 1 with highest values at week 60 in both strains (Table 1). IMPase 1 is the key enzyme of MI synthesis from glucose^26^, however, its regulation is not well understood and unknown in chicken. The simultaneous pattern in LSL hens suggested that there is a close relationship between hepatic MI concentrations and IMPase 1 content. The higher IMPase expression, the higher MI concentrations were in liver. However, the lowest hepatic MI concentration along variation in time was observed in week 24 (Table 1). At this week, IMPAse 1 had also higher concentrations compared to week 10. Besides being an egg component, MI is essential for an optimal ovary performance being involved in gonadotropin pathways promoting ovulation^27^; thus, at onset of egg laying or before week 24, the MI needs of the ovary increased strongly. Therefore, liver MI concentrations decreased and IMPase 1 expression was compensatorily adapted to increase MI synthesis to balance the requirement of fertility but without the capacity to maintain hepatic MI concentrations. Towards the end of the laying period in week 60, IMPase concentrations were well adapted but egg performance decreased and thereby, the sink for MI in the ovary was reduced resulting in higher hepatic MI concentrations.

**Table 1:** MI and IMPase 1 concentration in liver, kidney and muscle of LB and LSL hens in five periods of production.

	10 weeks	16 weeks	24 weeks	30 weeks	60 weeks	p-values
**MI (mg/g DM)**						
Liver						
LB	6.9 ± 0.68 7.2	6.6 ± 0.87	5.5 ± 0.67	6.2 ± 1.02	8.6 ± 0.47	Strain 0.09 Period < 0.01 Inter 0.51
LSL	± 0.56	5.3 ± 0.72	3.9 ± 0.64	5.3 ± 0.65	8.7 ± 0.60	
All	7.1^b^	6.0^bc^	4.7^c^	5.8^bc^	8.7^a^	
Muscle						
LB	1.1 ± 0.12	1.5 ± 0.26	1.3 ± 0.22	0.5 ± 0.12	1.9 ± 0.15	Strain 0.30 Period < 0.01 Inter 0.44
LSL	0.7 ± 0.14	1.5 ± 0.16	1.2 ± 0.22	0.5 ± 0.09	1.6 ± 0.17	
All	0.9^c^	1.6^ab^	1.3^bc^	0.5^d^	1.8^a^	
Kidney						
LB	8.5 ± 0.61	7.5 ± 0.54	7.1 ± 0.46	8.3 ± 0.88	9.5 ± 0.97	Strain 0.73 Period 0.08 Inter 0.62
LSL	8.2 ± 0.62	6.9 ± 0.67	8.2 ± 0.51	7.8 ± 0.69	9.1 ± 0.79	
**IMPase 1**^**2**^** (pg/mg protein)**						
Liver						
LB	0.08 ± 0.02	0.20 ± 0.03	0.17 ± 0.07	0.10 ± 0.02	0.32 ± 0.09	Strain 0.51 Period < 0.01 Inter 0.57
LSL	0.05 ± 0.01	0.15 ± 0.03	0.28 ± 0.08	0.18 ± 0.07	0.35 ± 0.08	
All	0.06^c^	0.18^bc^	0.23^ab^	0.14^bc^	0.33^a^	
Muscle						
LB	0.1 ± 0.01	0.07 ± 0.008	0.08 ± 0.006	0.06 ± 0.01	1.07 ± 0.07	Strain 0.47 Period < 0.01 Inter 0.34
LSL	0.12 ± 0.01	0.08 ± 0.007	0.11 ± 0.02	0.04 ± 0.01	0.96 ± 0.08	
All	0.11^b^	0.08^b^	0.09^b^	0.06^b^	1.02^a^	

Values for each variable are given as LSmeans ± SEM.

*MI*: *myo*-inositol, *IMPase 1* inositol monophosphatase 1.

Different letter indicates statistical differences along productive period of all hens. The number of replicates per strain and period was 10, with the exception of LSL hens at 60 weeks, where it was 9. Interactions between strain and period are indicated by “Inter.” p < 0.05.

In breast muscle, MI concentrations increased over time with highest values at week 60 which was paralleled by higher IMPase concentrations in liver. However, in muscle the lowest MI concentration along variation in time was observed in week 30 at peak of egg laying with out any compensatory increase in IMPase concentrations. Thus, muscle MI might be an additional source for supporting fertility, assuming that MI could be released from tissues into plasma and then, into ovary.

### Kidney *myo*-inositol and *myo*-inositol oxidase protein concentrations

Kidney MI concentrations did not vary over time and between strains (Table 1). As MI has been characterized to be an important organic osmolyte in mammal kidneys^28^, this result indicated that also in chicken a MI homeostasis in kidneys was established independently of productive period.

**Figure 1:**
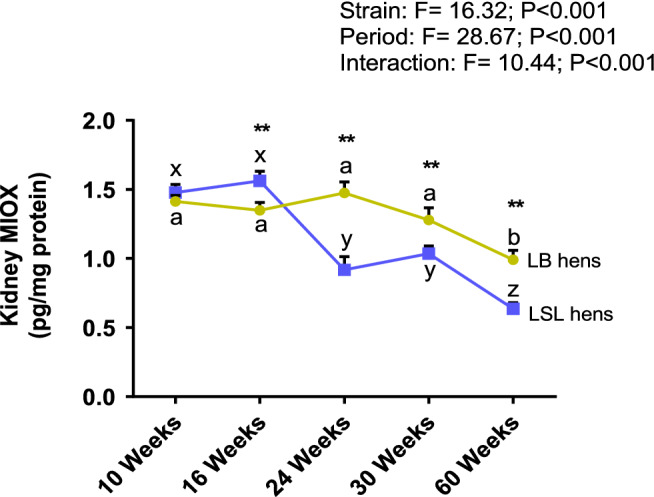
Concentrations of *myo*-inositol oxygenase (MIOX) in kidneys of LB (olive) and LSL (navy) hens at five productive periods. Symbols show mean ± SEM (n = 10 hens per strain and period). Two-way ANOVA followed by the post-hoc test Tukey HSD (Honestly Significant Difference) were used for statistical analysis. Different letters indicate significant differences within strain at different productive periods (p < 0.05) whereas **indicate highly significant differences (p < 0.01) between strains at the same productive period. F-values showed the ratio of between-groups to within-groups variances.

Kidney MIOX concentrations decreased with the progression of egg production, reaching its lowest value at week 60 (Fig. 1). MIOX concentrations were significantly lower in LB than in LSL hens at week 16 (1.6 ± 0.07 vs. 1.3 ± 0.05 pg/mg protein, respectively); however, LB hens had higher MIOX concentrations than LSL hens at weeks 24, 30, and 60 weeks (1.5 ± 0.08 vs. 0.9 ± 0.09, 1.3 ± 0.09 vs. 1.0 ± 0.05 and 1.0 ± 0.07 vs. 0.6 ± 0.04 pg/mg protein, respectively). Thus, a strong interaction occurred between the factors strain and period by the significantly stronger decline in MIOX protein expression in LSL hens at onset of egg laying.

In mammals, MI appeared to be catabolized through MIOX to d-glucuronic acid^29^. If reduced amount of enzyme reflected diminished activity, less MI degradation occurred in laying hens over time; however, this decrease was more pronounced in LSL hens. MIOX is the key enzyme for MI degradation so that this finding implicated that renal MI elimination may be less expressed in LSL hens during the whole egg laying period. Since this was not reflected by strain-related differences in MI concentrations and IMPase1 concentrations in tissues, either MI synthesis was higher due to higher activity of IMPase1 (activity was not determined) or MI absorption in the intestine was higher in LSL hens. In jejunum, brush border membrane-related phytase activities were with significantly lower in LSL than in LB hens^30^. This lower endogenous capacity to release MI from phytate in the digestive tract may result in less absorption of MI and therefore, to lower plasma MI concentrations. Plasma MI concentrations showed a significant interaction between strain and productive period with lower values in LSL hens at week 60^30^. Thus, lower MI availability might be compensated by reduced MIOX expression either to decrease degradation of MI (protecting MI content in plasma) or as response to lower substrate availability. A significant positive correlation (R^2^ = 0.37, p < 0.006) between MIOX expression and plasma MI concentrations at day 60 in both strains confirmed this assumption (data not shown). To sum up, MI metabolism in LSL hens was reflected by lower MI concentrations in plasma, and also by less MIOX expression. Furthermore, LSL hens had significantly lower MI concentrations in eggs, especially in the yolk, over the whole egg laying period compared to LB hens (− 13% in week 24, − 17.4% in week 30, − 15.7% in week 60)^20^. Due to the manifold biological functions of MI it can be concluded, that, since LSL hens were not able to maintain the MI homeostasis on the same level like the LB hens, this could have negative consequences for the efficiency of metabolism. Further research is needed to understand the biological meaning of this metabolite MI in chicken physiology.

### Metabolite profiles over time at different productive periods

**Figure 2:**
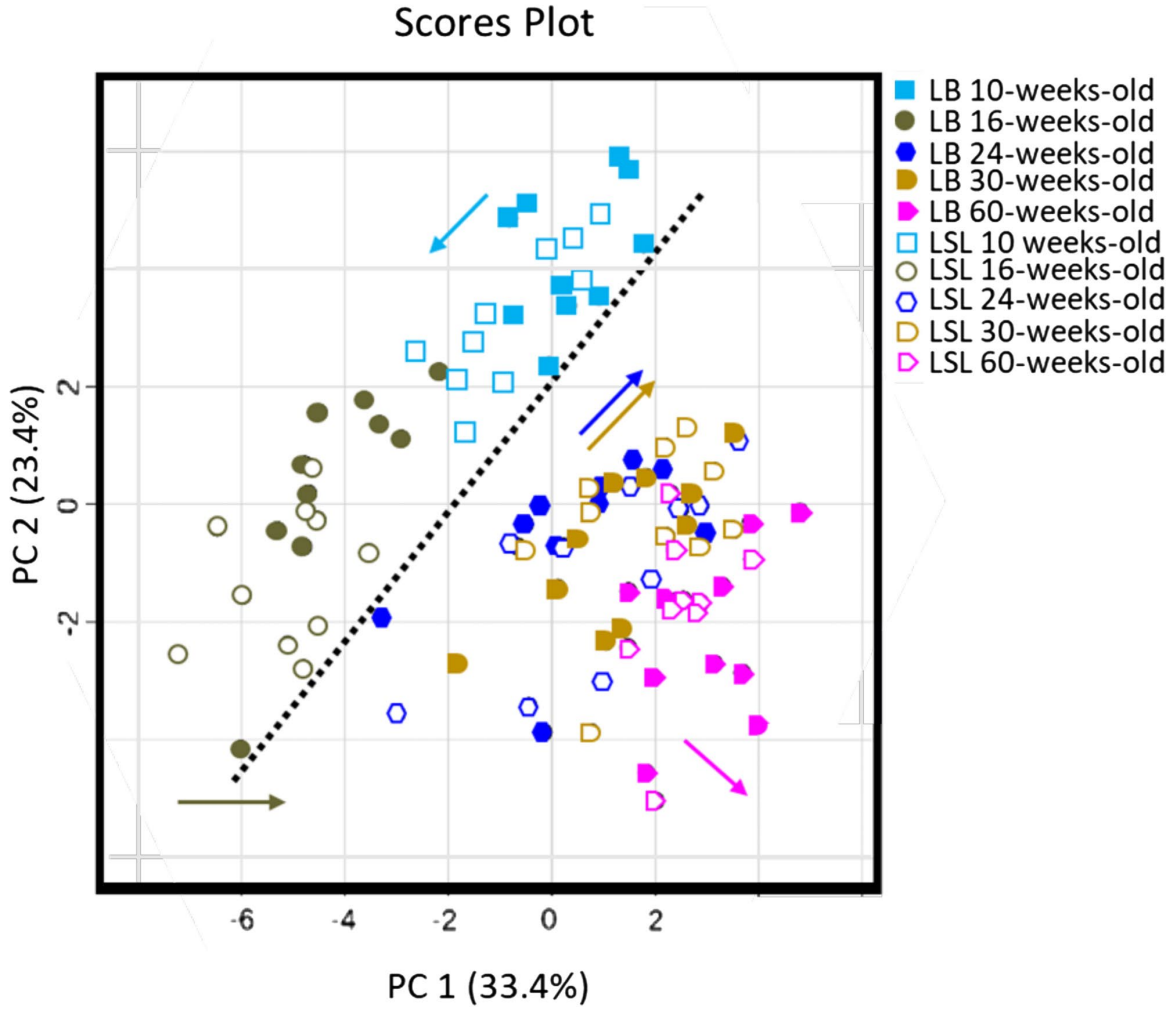
Principal component analysis (PCA) of plasma metabolite profiles of LB and LSL hens determined at five production periods. Each point indicates metabolite profile of one hen and each color indicates one specific period of production. Filled symbols indicate LB hens whereas open symbols indicate LSL hens. Dashed line indicates a clear separation between hens before and after laying-egg period. Color arrows show the direction of shifting according to each stage of production.

Metabolite profiling by targeted metabolomics approaches is a suitable tool to evaluate similarities and dissimilarities between strains and productive periods, respectively, in specific metabolic pathways related to energy and amino acid metabolism, mitochondrial function and inflammation. A principal component analysis (PCA) was used to reduce dimensionality of the big metabolomics data set by transforming the high number of variables into less. This is without loss of information but enable to identify differences between observations in general. Thus, each dot in the PCA represented one hen’s metabolite profile at a certain period. Overall, the metabolite profiles shifted from week 10 to week 16 along PC2 and between week 16 and 24, and week 30 and 60 along PC1 for both, LB and LSL hens (Fig. 2, Supplementary Table S1). According to this PCA, 56.8% of the accumulated variance is explained by the first two principal components.

Between weeks 16 and 24, egg laying started in both strains; thus, metabolic adaptation was likely to occur due to transition from growth to egg formation. Furthermore, the feed of laying hens was adjusted to the respective stage, thus observed differences could by also attributed to differences in diet composition; however, it remained unclear to what extent. Onset of egg laying is often associated with metabolic stress and inflammatory processes^31^. Therefore, the targeted metabolomics approach (IDQ p180 panel, Biocrates, Innsbruck, Austria) was used covering metabolites related to energy and amino acid metabolism, but also to oxidative stress and inflammation. It was aimed to characterize metabolic adaptation to onset of egg laying in LB and LSL hens by metabolite profiling. As a first evidence, the shifting in metabolite profiles changed direction in the PCA (Fig. 2; as indicated by the dotted black line) moving along the PC1. Metabolites of interest related to metabolic changes from week 16 to week 24 in each strain were identified by FDR adjusted P-value from t- student’s test (Tables 2, 3).

**Table 2:** LB hens—weeks 16 to 24: differential metabolite concentrations between weeks 16 and 24 in plasma according to FDR adjusted P-value from t-student’s test.

Metabolite (µmol/l)	Week 16	Week 24	FDR adjusted p-value	t-value_df_
**Amino acids**				
Tyrosine	337 ± 9.9	172 ± 7.3	< 0.001	13.09_18_
Threonine	557 ± 21.2	313 ± 20.1	< 0.001	7.70_18_
Tryptophan	144 ± 6.4	91.8 ± 4.74	< 0.001	6.77_18_
Histidine	262 ± 10.2	162 ± 16	< 0.001	5.41_18_
Asparagine	319 ± 14.1	210 ± 15	< 0.001	5.04_18_
Arginine	503 ± 10.7	389 ± 22.1	< 0.001	4.56_18_
Glutamine	1297 ± 35.7	883 ± 95.8	< 0.01	3.98_18_
Alanine	1156 ± 46.6	813 ± 63.7	< 0.01	3.96_18_
Lysine	474 ± 31.7	310 ± 38.5	< 0.01	3.96_18_
Proline	763 ± 29.2	592 ± 35.6	< 0.01	3.58_18_
**Biogenic amines**				
Carnosine	20.3 ± 1.4	7.5 ± 0.32	< 0.001	12.76_18_
trans-4-OH-Pro	134.4 ± 7.9	30.8 ± 3.6	< 0.001	11.44_18_
Alpha-AAA	2.1 ± 0.18	1.04 ± 0.1	< 0.001	5.35_18_
Sarcosine	21.6 ± 0.8	12 ± 1.57	< 0.001	5.32_18_
Spermine	0.58 ± 0.06	0.30 ± 0.01	< 0.001	4.99_18_
**Phospholipids**				
Sum of PCs	1862 ± 95.8	4728 ± 455	< 0.001	− 8.56_18_
Sum of lysoPCs	54.4 ± 3.38	19.3 ± 1.11	< 0.001	10.91_18_
Sum of SMs	237.4 ± 11.3	160.5 ± 14.2	< 0.01	4.15_18_

Comparison between LB hens at 16 (n = 10) and 24 (n = 10) weeks of age. Values are showed as means ± SEM.

*ADMA* asymmetric dimethylarginine, *Alpha-AAA* alpha-aminoadipic acid, *Met-SO* methionine sulfoxide, *PC* glycerophosphatidylcholines, *SM* sphingomyelins.

FDR-adjusted p < 0.05 as significance level was used. t-value indicates the ratio between the difference between and within both groups. Degrees of freedom (df) are shown in the figure as subscript of t-values.

**Table 3:** LSL hens—week 16 to 24: differential metabolite concentrations in plasma according to FDR adjusted p-value from t-student’s test.

Metabolite (µmol/l)	Week 16	Week 24	FDR adjusted p-value	t-value_df_
**Amino acids**				
Threonine	548 ± 21.6	275 ± 24.4	< 0.001	7.71_18_
Tryptophan	168 ± 6.3	89.3 ± 7.5	< 0.001	7.58_18_
Asparagine	347 ± 18.4	200 ± 12	< 0.001	7.11_18_
Histidine	260 ± 11.4	153 ± 10.5	< 0.001	6.58_18_
Glycine	1050 ± 32.3	689 ± 44.6	< 0.001	6.54_18_
Alanine	1090 ± 47.2	680 ± 44.7	< 0.001	6.29_18_
Tyrosine	289 ± 13.4	183 ± 13.1	< 0.001	5.73_18_
Proline	898 ± 50.4	536 ± 48.4	< 0.001	5.34_18_
Arginine	546 ± 18.1	408 ± 20.1	< 0.001	5.16_18_
Aspartate	72 ± 5.7	45 ± 3.5	< 0.001	4.44_18_
Serine	1071 ± 29.1	868 ± 42.4	< 0.01	3.96_18_
Valine	516 ± 21.7	348 ± 37.4	< 0.01	3.96_18_
Methionine	230 ± 12	165 ± 12.3	< 0.01	3.59_18_
Leucine	664 ± 29.3	516 ± 37.2	< 0.01	3.40_18_
Glutamine	1294 ± 42.7	1057 ± 65.8	< 0.01	3.26_18_
Isoleucine	291 ± 11.7	228 ± 20.0	< 0.05	3.03_18_
**Biogenic amines**				
trans-4-OH-Pro	134 ± 6.4	25 ± 1.79	< 0.001	20.48_18_
Sarcosine	24 ± 1.3	7.9 ± 0.74	< 0.001	10.92_18_
Carnosine	26.7 ± 1.2	10.2 ± 0.85	< 0.001	10.53_18_
ADMA	1.14 ± 0.05	0.75 ± 0.03	< 0.001	6.34_18_
Alpha-AAA	2.4 ± 0.2	1.2 ± 0.1	< 0.001	5.33_18_
Spermine	0.62 ± 0.05	0.36 ± 0.04	< 0.001	4.54_18_
Met-SO	19.9 ± 1	13.47 ± 1.32	< 0.01	4.07_18_
Kynurenine	0.44 ± 0.07	0.18 ± 0.02	< 0.01	3.73_18_
Taurine	774 ± 116	380 ± 49	< 0.01	3.17_18_
Putrescine	0.96 ± 0.1	1.69 ± 0.2	< 0.01	− 3.15_18_
Creatinine	2.95 ± 0.17	2.34 ± 0.2	< 0.05	2.41_18_
**Phospholipids**				
Sum of PCs	1951 ± 65.7	4726 ± 393	< 0.001	− 9.44_18_
Sum of lysoPCs	60.5 ± 4	19.9 ± 1	< 0.001	13.72_18_
Sum of SMs	246 ± 12.2	161 ± 12	< 0.001	4.88_18_
**Hexoses**				
Sum of hexoses	21,253 ± 998	17,995 ± 857	< 0.05	2.53_18_

Comparison between LSL hens at 16 (n = 10) and 24 (n = 10) weeks of age. Values are showed as means ± SEM.

*ADMA* asymmetric dimethylarginine, *Alpha-AAA* alpha-aminoadipic acid, *Met-SO* methionine sulfoxide, *PC* glycerophosphatidylcholines, *SM* sphingomyelins.

FDR-adjusted p < 0.05 as significance level was used. t-value indicates the ratio between the difference between and within both groups. Degrees of freedom (df) are shown in the figure as subscript of t-values.

### Metabolite profile differences associated with egg laying period

**Figure 3:**
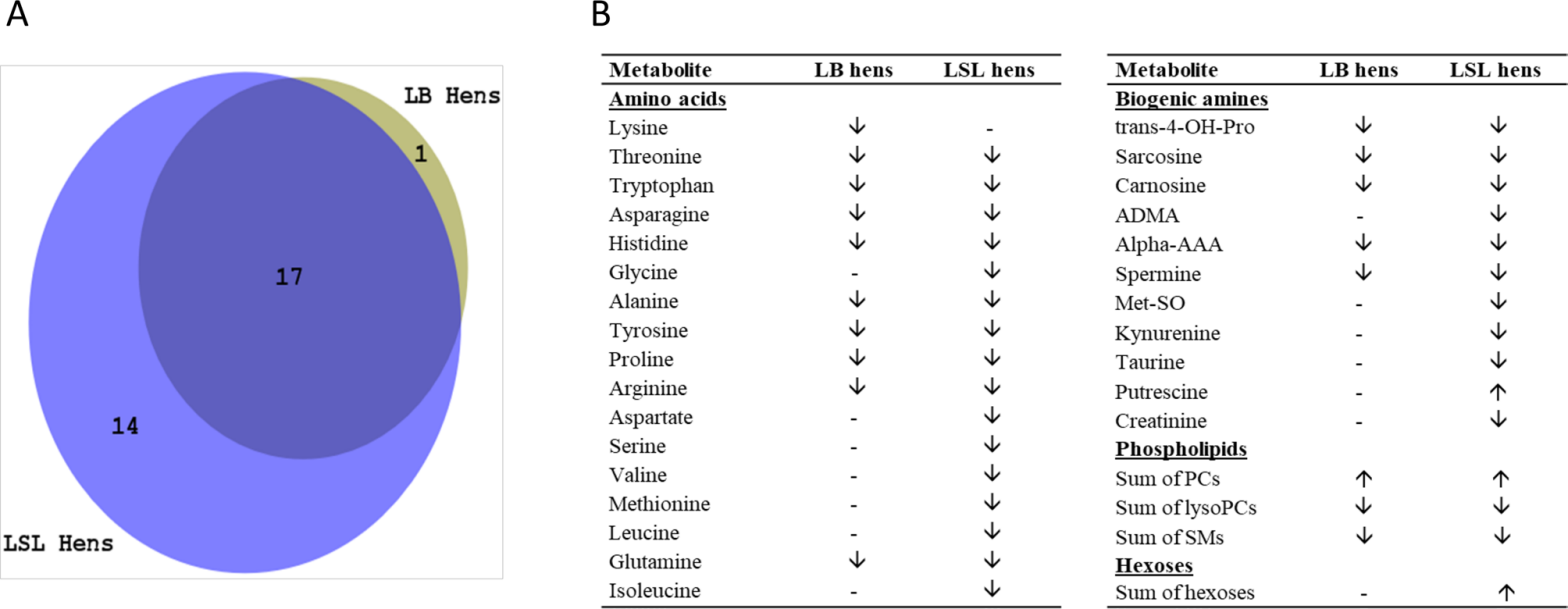
(**A**) Venn diagram indicating the number of plasma metabolites expressed differently between 16 and 24 weeks old LB (olive) and LSL (navy) hens. (**B**) List of the metabolites depicted in the Venn diagram. The symbols “↑” and “↓” indicate that metabolite expression increased or decreased at week 24 in comparison to week 16. Symbol “–” indicates this metabolite did not vary in one strain. Differences were revealed by FDR adjusted p-value from t-student’s test (< 0.05). *ADMA* asymmetric dimethyl arginine, *Alpha-AAA* alpha amino adipic acid, *Met-SO* methionine sulfoxide, *PC* phosphatidylcholines, *SM* sphingomyelins.

Plasma metabolite concentrations of LB and LSL hens were affected by the onset of egg laying (16 vs. 24 weeks) (Tables 2, 3, respectively) with some specific conditions in the two strains (Fig. 3). A total of 17 metabolites changed in the same direction in both strains over time (Fig. 3A). While the sum of PCs increased, the sum of lysoPCs and SMs decreased from week 16 to 24. The biological meaning of glycerophospholipids in chicken metabolism is quite unknown. Phospholipids have been reported to be essential components of egg yolk^32^. Elevations in the sum of plasma PCs at week 24 indicated increased PCs biosynthesis required for egg yolk phospholipids because PCs are essential components of egg yolk, comprising 45–80% of total phospholipids^32^. The reduction of lysoPCs and SMs in plasma may be related to inflammatory processes in tissues such as the reproductive tract due to start of laying. LysoPCs were sources of long-chain fatty acids, often unsaturated ones, which served as substrate for macrophages membrane remodeling in times of an inflammatory response^33^. A higher membrane fluidity then enabled rapid invasion of tissues by the macrophages. Onset of egg laying is most likely associated with local tissue transformation and thereby inflammation, especially in liver and oviduct of the hen. Sphingolipids such as SMs and their derivatives served as precursors of inflammatory signals in cells^34^. At onset of egg laying, cells, especially hepatocytes, may release less SMs into plasma because these metabolites are needed for cellular pathways related to an increase in energy and substrate metabolism. Furthermore, several amino acids such as Thr, Trp, Asn, His, Ala, Tyr, Pro, Arg, Gln and biogenic amines and amino acid derivatives such as trans-4 OH Pro, sarcosine, carnosine, alpha amino adipic acid (alphaAAA) and spermine were lower in week 24 than 16 in both strains (Fig. 3B). Most likely, egg formation needed amino acids not only for egg proteins but also for several bioactive substances such as anti-oxidant and anti-inflammatory molecules^35^; thus, concentrations of some of the amino acids and biogenic amines decreased. However, as mentioned before, also the change of feed to meet the nutritional requirements of the hen at specific productive periods may have also contributed to changes in metabolites such as amino acids.

A total of 15 metabolites were differently expressed at weeks 16 and 24 only in one of the strains, 1 metabolite in LB and 14 in LSL hens (Fig. 3A,B). In LB hens, the Lys concentration was lower in week 24 but not in LSL hens. Lys is an essential amino acid in laying hens determining the protein synthesis rate in liver and oviduct^36^. Because both strains were provided with the same feed, results indicated differences in Lys utilization for egg production between the strains.

Metabolites which were lower at week 24 only in LSL hens were amino acids, Gly, Asp, Ser, Val, Met, Leu, Ile, and biogenic amines, asymmetric dimethyl arginine (ADMA), methionine sulfoxide (Met-SO), kynurenine, taurine, creatinine. In general, it is difficult to interpret these findings in detail, but they confirm that the two laying strains handled onset of egg laying differently. Most interesting, all branched chain amino acids (BCAA), Leu, Ile and Val, were lower in week 24 compared to week 16 while hexoses and the biogenic amine putrescine were higher in LSL hens only. BCAA are well-known metabolic regulators in mammalian species via the mammalian target of rapamycin (mTOR) pathway; furthermore, high plasma BCAA concentrations were associated to patho-physiologies such as diabetes, insulin resistance, pro-inflammation and oxidative stress^37,38^. In female broiler chickens, an analogous relationship was observed. Low BCAA concentrations inhibited fatty acid synthesis and enhanced fatty acid oxidation in the liver via mTOR pathway^39^. Therefore, LSL hens might have a more efficient fatty acid energy utilization compared to LB hens. Biogenic amines and derivatives of amino acids are modulating many pathways related to energy balance and metabolic functions, but also to oxidative stress and inflammation. Amongst them, ADMA, which was lower in week 24 in LSL hens only, is well-known for its role in promoting endothelial dysfunctions^40^, thus decreased concentrations after onset of egg laying may be beneficial for LSL hens. Furthermore, the manifold biological role of kynurenine indicated that, since it is lower in week 24 only in LSL hens, strong differences in metabolism existed between both strains although they did not differ in laying performance. Kynurenine belongs to the Trp metabolites, which signal to various cells of the body including microbiota and control systemic energy metabolism, immune cell functions and adipose tissue metabolism^41^. To conclude, the metabolomics approaches allows for generating novel hypotheses about metabolic regulation and, more research is needed in poultry to test those hypotheses and better understand physiology of egg laying performance in different strain.

### Association of MI metabolism with plasma metabolites

**Figure 4:**
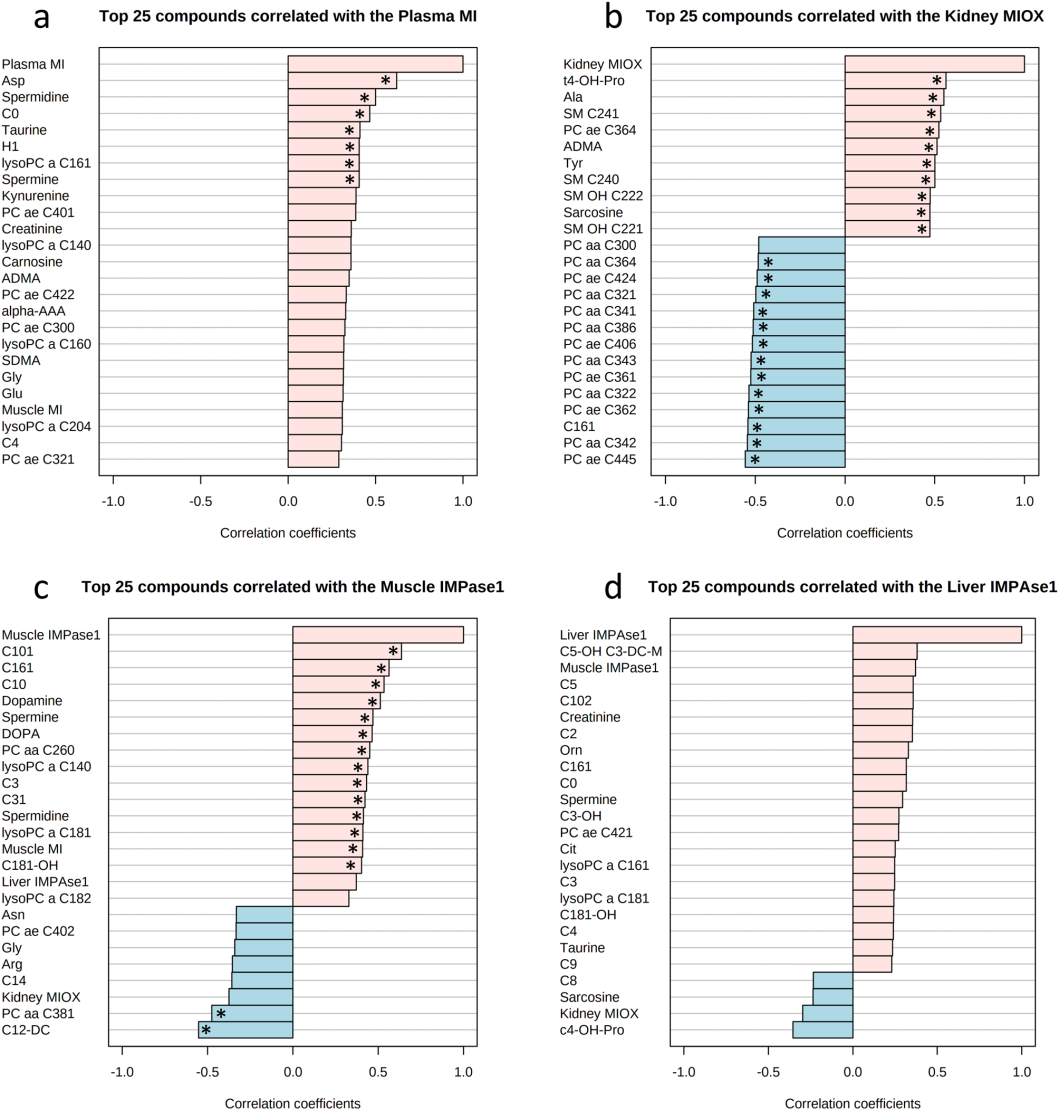
Pearson’s correlations using pattern search function in MetaboAnalyst 4.0 between components of MI metabolism and plasma metabolites in all hens and all time points. (**a**) Correlation to plasma MI; (**b**) correlation to kidney *myo*-inositol oxygenase (MIOX); (**c**) correlation to muscle inositolmonophosphatase 1(IMPase 1); (**d**) correlation to liver IMPase 1. Correlations with Pearson’s correlation coefficient (r) ≥ 0.4 (*) were discussed in detail in the text; corresponding statistical information is provided in Supplementary Table S1. *Ala* alanine, *Alpha-AAA* alpha-aminoadipic acid, *Arg* arginine, *Asn* asparagine, *Asp* aspartate, *C0* carnitine, *ADMA* asymmetric dimethylarginine, *Ala* alanine, *C3* propionylcarnitine, *C3:1* propenoylcarnitine, *C4* butyrylcarnitine, *C10* decanoylcarnitine, *C10:1* decenoylcarnitine, *C10:2* decadienylcarnitine, *C12-DC* dodecanedioylcarnitine, *C14* tetradecanoylcarnitine, *C16:1* hexadecenoylcarnitine, *C18:1-OH* hydroxyoctadecenoylcarnitine, *DOPA* dihydroxyphenylalanine, *Gly* glycine, *Glu* glutamate, *H1* sum of hexoses, *IMPase 1 myo*-inositol monophosphatse 1, *Lyso PC* lysophosphatidylcholines, *Met* methionine, *Met-SO* Methionine sulfoxide, *MI myo*-inositol, *MIOX myo*-inositol oxygenase, *PC* phosphatidylcholine, *SDMA* symmetric dimethylarginine, *SM* sphingomyelins, *t4-OH-Pro* trans-4-hydroxyproline, *Tyr* tyrosine.

Correlation analyses between components of MI metabolism and plasma metabolite in all hens irrespective of strain and periods revealed relationships, which were only considered for discussion when Pearson correlation coefficient (r) was ≥ 0.5 as high-strong association and ≥ 0.4 as medium-strong association. Although a linear association is not proving causal relationship, correlation analyses were used to create new hypotheses about the relevance of MI metabolism for systemic metabolism in laying hens. Figure 4 demonstrates associations of plasma metabolite concentrations (TOP25) with plasma MI (Fig. 4a), kidney MIOX (Fig. 4b), muscle IMPase 1 (Fig. 4c) and liver IMPase 1 concentrations (Fig. 4d). Statistical evaluation of correlations is given in Supplementary Table S2.

Plasma MI was positively high-strong associated with aspartate, spermidine and carnitine and medium-strong associated with taurine, hexoses, lysoPC a C16:1 and spermine. Orally applied d-aspartate lowered body temperature and plasma triglycerides, while plasma glucose increased, suggesting that thereby, energy metabolism was modulated by d-aspartate in broilers^42^. However, it is unclear to which proportion d- and l-enantiomers of this amino acid exist in plasma of laying hens. Spermidine and also spermine belong to the biogenic amines which have multiple biological functions such as improving glucose homeostasis, insulin sensitivity, and reducing adiposity and hepatic fat accumulation^43^. The amino acid taurine belongs to the free amino acid pool in tissues with highest concentrations in heart muscle; its biological meaning is defined as protective against cardiomyopathy. Higher plasma concentrations indicated cardiac health and well-function^44^. For a long time, carnitine is known to improve oxidative energy metabolism by improving the long-chain fatty acid transport into mitochondria for beta-oxidation and oxidative phosphorylation to generate ATP energy^45^. Finally, lyso-PCs decreased under inflammatory condition providing unsaturated fatty acids for macrophages, thereby ameliorating mobility of these immune cells to migrate into tissues. High lysoPC concentrations in plasma indicated an anti-inflammatory condition^46^. To summarize, plasma MI is correlated with several metabolites which indicate an improved energy metabolism and anti-inflammation in laying hens suggesting an important and positive meaning of MI in chicken metabolism.

MIOX concentrations were positively high-strong associated with trans-4-hydroxyproline, alanine, SMs (C24:1, C24:0), SMs OH (C22:2, C22:1), PCs (ae C36:4), asymmetric dimethylarginine (ADMA), tyrosine, sarcosine and methioninesulfoxide (Fig. 4B, Table S1). Positive medium-strong associations of MIOX was observed with methionine, symmetric dimethylarginine (SDMA), lysoPC C18:0 and hexoses. Kidney MIOX was negatively high-strong associated with acyl-acyl and acyl-ether PCs (for details see Table S1) and acylcarnitine C16:1; and negative medium-strong associations were observed with further acyl-acyl and acyl-ether PCs (see Table S1 for details) and acylcarnitines (C10:2, C3). Dimethylarginines were assessed to be toxic non-proteinogenic amino acids derived from proteolysis, which inhibited nitric oxide production and thereby, were promoting metabolic dysfunctions^47^. Positive associations of renal MIOX concentrations with dimethylarginines could hint to a relationship of MI degradation with proteolysis as cause or as consequence of a more tensed metabolism. Amino acids derivatives, trans-4-hydroxyproline and methionine sulfoxide, belong to the anti-oxidative system protecting against mitochondrial dysfunction and concomitant reactive oxygen species (ROS) production. Higher concentrations of these derivatives were observed in plasma of human patients with heart failure and hypoxaemia compared to healthy controls^48^. Thus, high MIOX expression might be related to an imbalance in oxidative metabolism in laying hens. This hypothesis is supported by the finding that upregulated renal MIOX is disrupting mitochondrial integrity by increased mitochondrial fragmentation, mitophagy, apoptosis and ROS production in diabetic kidney disease mouse model and cell culture cells, respectively^49^. The relationship with phosphatidylcholines and sphingomyelins are difficult to interpret in all correlation approaches; further research is needed to understand the biological meaning of lipids in metabolic regulation of chicken.

Muscle IMPase 1 concentrations were positively high-strong associated with acylcarnitines (C10:1, C16:1, C10), dopamine, spermine and 1-3,4-dihydroxyphenylalanine (DOPA). Positive, medium-strong associations were observed with PC aa C26:0, lysoPCs (C14:0, C18:1), acylcarnitines (C3, C3:1, C18:1-OH), spermidine and muscle MI. Muscle IMPase 1 was negatively high-strong associated with acylcarnitine C12-DC and PC aa C38:1. Acylcarnitines (AC), especially the long-chain AC, are derived from fatty acid oxidation in mitochondria. They can be released into plasma to serve as easy-to-use energy substrates for other tissues and reflect a high mitochondrial activity but an imbalance between acetyl-CoA production and use in the tricarbonic acid cycle^50^. Thus, acylcarnitines were released into plasma as intermediary products and thereby, mitochondria were protected against lipotoxicity^51^. High IMPase 1 concentration, if associated with high IMPase 1 activity, generates MI which in turn stimulated mitochondrial function, especially fatty acid oxidation^52^. A close relationship between muscle IMPase 1 and plasma DOPA and dopamine, respectively, suggested a connection of MI metabolism with the neuroendocrine system. This hypothesis is confirmed by the finding that dietary MI supplementation increased dopamine and serotonin concentrations in broilers^14^.

Correlations of plasma metabolites with liver IMPase 1 were only weak, thus they were not considered in this context.

### Metabolite profile and performance

**Figure 5:**
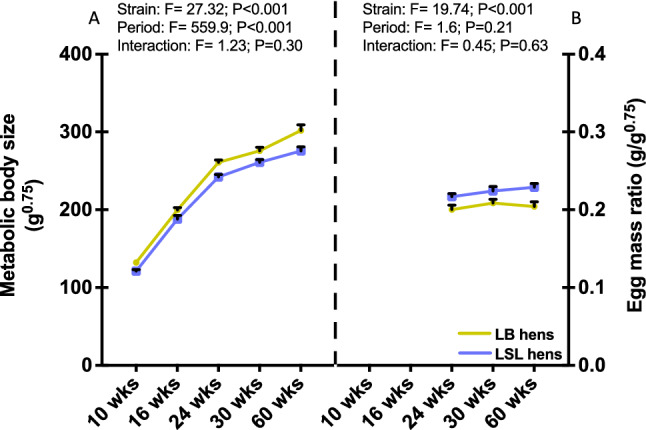
Metabolic body size (left y-axis) (**A**) and egg mass/metabolic size ratio (right y-axis) (**B**) of LB (olive) and LSL (navy) hens at five and three productive periods, respectively. Bars show means ± SEM (n = 10 hens per strain and period). Two-way ANOVA followed by the post-hoc test Tukey HSD (honestly significant difference) were used for statistical analysis. F-values showed the ratio of between-groups to within-groups variances.

According to the findings of this study, LSL and LB hens differed in adaptation to onset of egg laying despite equal feed intake. As major results, LSL hens expressed lower body weight but similar average daily gain compared to LB hens throughout the trial^30^; consequently, the metabolic body size (body weight (g)^0.75^) was also lower (Fig. 5A). Performance expressed as g egg mass/g metabolic body size was higher in LSL hens (Fig. 5B). LSL hens had a lower MIOX expression in kidney and lower BCAA, methionine sulfoxide, asymmetric dimethylarginine and kynurenine concentrations after onset of egg laying. The former might indicate less muscle protein turnover due to the lower body weight, while lower kynurenine and methionine sulfoxide pointed to a less stressed metabolic condition in LSL hens despite their marginally higher performance.

MI metabolism appeared to be associated with energy metabolism and immune functions. The higher MI availability, the lower the risk for metabolic disturbances. The impact of novel indicators found in this study for control of optimal raising and feeding conditions of laying hens needs to be evaluated to improve health and performance.

## Materials and methods

### Hens and diets

The study was carried out in compliance with the ARRIVE guidelines^53^. Experimental design and management procedures were approved by the ethics committee of the Regierungspräsidium Tübingen following the German animal welfare regulations (Project no., HOH50/17TE). A detailed description of the entire experiment was provided by a previous study^30^. In short, 50 Lohmann Brown-Classic (LB) and 50 Lohmann LSL-Classic (LSL) hens with distinct genetic background were used. All hens were from the same hatch and raised using the same conditions for both strains. A 2 × 5-factorial arrangement of treatments was used by using hen strain and 5 periods of production as factors (10, 16, 24, 30, and 60 weeks of age). Initially, all hens were housed together on deep litter bedding. Ten days before each sampling, 10 hens per strain were randomly chosen and kept individually in metabolism units (1 m^3^) in a randomized block design. The room temperature was set to 18–22 °C during sampling periods. All nutrients were calculated according to the recommended levels (Lohmann Tierzucht GmbH) for each production stage. Diets were based on corn and soybean meal to ensure minimum plant intrinsic phytase activity. Feed and tap water were provided for ad libitum consumption. On the day of sampling, the 20 hens were anesthetized by a gas mixture of 35% CO_2_, 35% N_2,_ and 30% O_2_ and immediately decapitated. Body weights were determined at the end of and egg mass was measured during the excreta-collection periods in week 24, 30 and 60 of the trial and data are already published^30^. However, to discuss these data in the context of the metabolite profiles, the mean metabolic body size (± SEM; g^0.75^) was calculated for each strain and time period, and ratio of egg mass/g metabolic body size was calculated.

### Plasma and tissue sampling

Following decapitation, trunk blood was collected in EDTA tubes and then centrifuged at 2490 × *g* for 10 min at 6 °C (Megafuge 2.0 R, Thermo Fisher Scientific, Waltham, MA). Subsequently, circa 500 µl of plasma was collected in 2 ml tubes, shock-frozen in liquid nitrogen, and stored on dry ice. Hens carcasses were eviscerated. Breast muscle and medial sections of the liver and kidney were sampled. All organ samples were washed in 1× PBS, cut into small pieces, shock-frozen into liquid nitrogen, and collected in pre-chilled cryotubes. Finally, plasma and organ samples were transported on dry ice to the lab and stored at − 80 °C for further analysis.

### Tissue homogenization

Liver, kidney, and breast muscle samples were ground by using a mortar and a pestle chilled with liquid nitrogen. 420 mg of ground tissue was mixed with 500 µl 1× PBS plus protease inhibitor (Complete mini, Hofmann-La Roche, Mannheim, Germany) in lysing matrix tubes (5076-400, MP Biomedicals, France) containing silica beads. Subsequently, tissues were homogenized by using Fast-prep homogenizer (FastPrep^®^-24 5G, MP Biomedicals, China) at 6 m/s for 30 s 3×. Upon homogenization, tissues were centrifuged for 15 min at 1500×*g* (Centrifuge 5424R Eppendorf, Germany). Supernatants were stored at − 80 °C until further analysis.

### Protein quantification

Tissue homogenates were diluted at 1:400 in distilled water for liver, kidney, and breast muscle. Protein concentrations were determined by the method according to Bradford (Bradford Reagent, SERVA, Heidelberg, Germany) in triplicate.

### *Myo*-inositol determination in liver, kidney and breast muscle

*Myo*-inositol concentrations were measured by a commercially available spectrophotometric kit (K-INOSL 02/14, Megazyme International, Ireland,) in tissue homogenates previously diluted at 1:120 for liver and kidney and at 1:40 for muscle in distilled water. The K-INOSL assay was down-scaled to 96 microtiter plates (655101, Greiner bio-one, Germany), and eight samples were run per assay. All samples were assessed in duplicate, and concentrations were calculated according to the standards provided by the kit. Final values were normalized by tissue dry matter (DM) to get a final unit of mg MI/g DM.

### Inositol monophosphatase 1 and *myo*-inositol oxygenase expression

Concentrations of kidney MIOX as well as liver and muscle IMPase 1 were measured by using commercial enzyme-linked immunoassay kits (Chicken MIOX ELISA Kit, MBS7215577 and Chicken IMPA1 ELISA Kit, MBS7235623, Mybiosource). Intra- and inter-assay coefficients of variation were reported as 5.5% and 7.3%, respectively. Protocols were performed according to manufacturer´s guidelines. In brief, aliquots of 100 µl from liver, kidney and muscle homogenates (containing in average (± SEM) 60.6 ± 7.8, 56.7 ± 7.5 and 120.6 ± 4.5 mg/ml protein, respectively) were buffered with balance solution (provided by the kit) and incubated for 1 h at 37 °C together with anti-IMPase and anti-MIOX antibodies conjugated with horseradish peroxidase (HRP). The plates were washed manually 5 times and incubated with the substrate for HRP. After 15 min, a blue colored complex was formed, and a stop solution was added to end the reaction, creating a yellow color. The colorimetric intensity was measured at 450 nm using a microplate reader (Infinite^®^ Infinite M Nano, TECAN, Austria). MIOX and IMPase 1 concentrations were extrapolated from the standard values by a 5 parameter logistic (5-PL) curve-fit (Magellan software, Tecan GmbH 2016, Austria). All values were normalized against tissue homogenate protein concentration (pg enzyme/mg total protein).

### Targeted metabolomics approach

Plasma metabolite profile analysis was performed by using the AbsoluteIDQ p180 Kit (Biocrates Life Science AG, Innsbruck, Austria) by of Biocrates Life Science AG. Briefly, AbsoluteIDQ p180 Kit was created for identifying up to 188 metabolites belonging to amino acids, biogenic amines, acylcarnitines, phosphatidylcholines, lysophosphatidylcholines, sphingolipids, and hexoses. The assay was based on phenylisothiocyanate (PITC) derivatization in the presence of internal standards followed by FIA-MS/MS (acylcarnitines, (lyso-) phosphatidylcholines, sphingomyelins, hexoses) and LC–MS/MS (amino acids, biogenic amines) using a SCIEX 4000 QTRAP^®^ (SCIEX, Darmstadt, Germany) or a Xevo TQ-S Micro (Waters, Vienna, Austria) instrument with electrospray ionisation (ESI). The experimental metabolomics measurement technique is described in detail by patent US 2007/0004044^54^. All pre-analytical and analytical procedures were performed, documented, and reviewed according to the ISO 9001:2008 certified in-house quality management rules and guidelines of Biocrates Life Sciences AG.

### Statistical analyses

#### Parametric statistics

Variance components estimation from MI and key MI enzymes from all the production periods were performed by using restricted maximum likelihood (REML) using Kenward and Roger as the method to determine degrees of freedom (_df_). Least square (LS) means comparison between hen lines and productive stages were analyzed by using mixed model procedures (SAS version 9.4, SAS Institute Inc., Cary, NC). For this experiment the following model was used: Y_ijklm_ = μ + α_i_ + β_j_ + (αβ)_ij_ + γ_k_ + (γβ)_kj_ + δ_l_ + ϕ_m_ + ε_ijklm_, where Y_ijklm_ = response variable, μ = overall mean, α_i_ = effect of strain (fixed), β_j_ = effect of period (fixed), the interaction between strain and period (fixed), γ_k_ = block (random), the interaction between block and period (random), δ_l_ = metabolism unit (random), ϕ_m_ = father/rooster (random), and ε_ijklm_ = residual error. Data were tested and confirmed to be normally distributed by the use of the D’Agostino and Pearson omnibus normality test. Values for the table were given as LSmeans ± SEM. Different letters indicates significant differences between productive period (p < 0.05), whereas * or ** indicates difference (p < 0.05) and high difference (p < 0.01), respectively, between LB and LSL hens at a specific production period.

#### Metabolomics data analyses and visualization

Plasma metabolite concentrations from each hen were provided in µmol/l by Biocrates as original data. Metabolomics data were analyzed and visualized by using MetaboAnalyst 4.0^55^. Briefly, metabolite concentrations from all the hens were normalized by generalized logarithmic transformation, mean-centered, and divided by the square root of the standard deviation of each variable (Pareto scaling). Phosphatidylcholines (PCs), lysophosphatidylcholines (LysoPCs) and sphingomyelins (SMs) were summed and analyzed as the sum of PCs, sum of lysoPCs, and sum of SMs, respectively. Principal component analysis (PCA) as an unsupervised method was used to display differences in metabolic profiles between stage of productions and between hen strains. Once a change in metabolite profiles was identified and selected, False Discovery Rates (FDR) adjusted p-values from t-student’s test were performed to identify which metabolites caused the variation in metabolite profile within each strain. Depiction of the number of differential metabolites were performed by the web application BioVenn^56^. Correlation analyses of linear associations were done using Pearson’s correlation (MetaboAnalyst 4.0). Comparisons and visualization between LB and LSL were made by using unpaired t-student’s test (GraphPad Prism version 6.07, La Jolla, CA, USA). Values for tables and figures were given as student’s t-test means ± SEM. Different letters indicated significant differences over productive period (FDR-adjusted p or p < 0.05).

## Supplementary Information

Supplementary Information.

## Data Availability

The datasets generated during and/or analyzed during the current study are not publicly available due to lack of an appropriate repository for animal metabolomics data but are available from the corresponding author on reasonable request.
