# Supplementary material for: Interrelationship of myo-inositol pathways with systemic metabolic conditions in two strains of high-performance laying hens during their productive life span
Source: Sci Rep. 2021 Feb 25;11:4641. doi: 10.1038/s41598-021-84169-x (PMC7907342; doi:10.1038/s41598-021-84169-x)
Supplement: Supplementary file 1 — Supplementary Information. [file 41598_2021_84169_MOESM1_ESM.docx]

**Interrelationship of myo-inositol pathways with systemic metabolic conditions in two strains of high-performance laying hens during their productive life span**

Fernando Gonzalez-Uarquin^1^, Vera Sommerfeld^1^, Markus Rodehutscord^1^ and Korinna Huber^1*^

^1^Institute of Animal Science, University of Hohenheim, 70599 Stuttgart, Germany.

*Corresponding author: [korinna.huber@uni-hohenheim.de](mailto:korinna.huber@uni-hohenheim.de)

**Supplementary Information**

Supplementary Table S1

Supplementary Table S2

**Table S1.** **Concentration of amino acids, biogenic amines, acylcarnitines, glycerophospholipids, sphingomyelins and hexoses in plasma of LB and LSL hens in five productive periods.**

| **Metabolite**  (µmol/l) | **Strain** | **Week 10** | | | | | | | | | | **Week 16** | **Week 24** | **Week 30** | **Week 60** |
| --- | --- | --- | --- | --- | --- | --- | --- | --- | --- | --- | --- | --- | --- | --- | --- |
| **Amino acids** |  |  | | | | | | | | | |  |  |  |  |
| Alanine | LB  LSL | 815±125  951±142 | | | | | | | | | | 1156±147  1090±149 | 813±201  680±141 | 706±190  566.5±138 | 452.2±112  442.8±78.6 |
| Arginine | LB  LSL | 345±38.7  390±41.8 | | | | | | | | | | 503±34.0  546±57.2 | 389±70.1  408±63.5 | 360.8±43.4  359.8±38.3 | 273.6±46.8  330±32.7 |
| Asparagine | LB  LSL | 184±30.0  213±39.0 | | | | | | | | | | 319±44.6  347±58.3 | 210±47.5  200±38.1 | 222.2±25.9  220.1±32.8 | 166±50.1  175±33.6 |
| Aspartate | LB  LSL | 36±5.90  42±7.10 | | | | | | | | | | 65.1±20.4  72.4±18.1 | 56.4±14.3  44.9±11.1 | 49±10.7  43.9±11.2 | 73.6±34.8  53±24.9 |
| Citrulline | LB  LSL | 5.98±1.34  7.83±1.63 | | | | | | | | | | 8.38±1.49  10.25±2.06 | 7.18±2.60  13±3.90 | 7.44±2.39  10.14±2.60 | 11.5±3.25  9.53±1.90 |
| Glutamine | LB  LSL | 960±137  1102±128 | | | | | | | | | | 1297±113  1294±135 | 883±303  1057±208 | 777.6±161  875.5±100 | 749.2±165  953.2±155 |
| Glutamate | LB  LSL | 192.7±22.4  280.7±43.5 | | | | | | | | | | 300±70.8  400±107 | 354.7±76.2  338±62.0 | 252.3±39.7  268.3±46.5 | 287.2±42.1  303.8±69.0 |
| Glycine | LB  LSL | 607.4±65.5  706.6±83.1 | | | | | | | | | | 834±90.6  1049.8±102 | 786.7±156  689±141 | 744.7±85.9  674.8±115 | 574±100  542.4±61.70 |
| Histidine | LB  LSL | 199.1±30.1  188.6±40.4 | | | | | | | | | | 262±32.3  260.3±36.2 | 162±50.6  153±33.2 | 160.5±35.7  146.7±19.1 | 140.1±20.5  142.3±14.6 |
| Isoleucine | LB  LSL | 163.2±20.6  184.6±22.6 | | | | | | | | | | 263.6±39.7  291±37.0 | 234.8±40.8  228±63.9 | 224.5±43.2  207.3±45.4 | 163±35.5  170.6±25.1 |
| Leucine | LB  LSL | 375.9±39.0  425.1±40.2 | | | | | | | | | | 607.6±96.8  664.4±92.7 | 551±74.1  516.2±118 | 487.1±85.0  459.8±85.3 | 383.8±54.7  390±34.6 |
| Lysine | LB  LSL | 271.8±84.0  239±85.10 | | | | | | | | | | 474±100  563.8±141 | 310±121.7  410±185.5 | 261.7±53.6  323.5±94.4 | 171.2±33.8  259.6±97.8 |
| Methionine | LB  LSL | 224.7±37.5  188.7±30.5 | | | | | | | | | | 280.6±46.7  229.6±38.2 | 238.5±80.2  165.4±39.0 | 250.1±48.2  184.7±70.3 | 161.7±33.0  136.8±26.1 |
| Ornithine | LB  LSL | 65.4±32.5  60.3±19.70 | | | | | | | | | | 173.6±61.8  238.2±75.9 | 223.8±58.3  204.6±67.1 | 245.2±54.0  237±57.5 | 170.8±73.6  234±53.0 |
| Phenylalanine | LB  LSL | 153.5±11.5  158±13.6 | | | | | | | | | | 196.7±22.5  181.7±29.1 | 182.3±25.4  163.1±28.2 | 170.7±27.0  146.3±20.4 | 135.1±15.2  130.3±7.60 |
| Proline | LB  LSL | 480.4±90.1  586.6±89.1 | | | | | | | | | | 763±92.5  898±159 | 592±113  536±153 | 610±91.4  547.9±99.8 | 477.8±120  425.5±70.5 |
| Serine | LB  LSL | 714.9±75.2  923±83.56 | | | | | | | | | | 863.4±88.4  1071±92.0 | 765.8±191  868.1±134 | 757±82.3  884.4±46.5 | 716±99.87  795.5±107 |
| Threonine | LB  LSL | 309.4±85.9  340.6±49.1 | | | | | | | | | | 557±67.1  548±68.5 | 313±63.6  275±77.2 | 368.7±64.2  333.7±39.3 | 328±103  327±86.5 |
| Tryptophan | LB  LSL | 101±9.40  105.3±8.76 | | | | | | | | | | 144±20.4  168±20.1 | 91.8±15.0  89.3±24.0 | 98.8±12.3  94.1±13.6 | 78.3±8.15  85.6±14.6 |
| Tyrosine | LB  LSL | 287.3±46.8  297±37.4 | | | | | | | | | | 337.1±31.5  289±42.3 | 172±23.2  183.2±41.5 | 160.5±40.2  156.3±18.4 | 119±18.2  140.1±19.6 |
| Valine | LB  LSL | 230.3±40.5  284.6±45.2 | | | | | | | | | | 430.3±88.0  516±68.8 | 357.1±59.7  384±118 | 351.7±78.2  325.4±76.5 | 273.3±68.1  301±52.86 |
| **Metabolite**  (µmol/l) | **Strain** | **Week 10** | | | | | | | | | | **Week 16** | **Week 24** | **Week 30** | **Week 60** |
| **Biogenic amines** |  |  | | | | | | | | | |  |  |  |  |
| ADMA | LB  LSL | 0.76±0.09  0.84±0.08 | | | | | | | | | | 1.01±0.15  1.14±0.18 | 0.88±0.16  0.75±0.11 | 0.64±0.10  0.61±0.11 | 0.56±0.04  0.54±0.09 |
| alpha-AAA | LB  LSL | 0.99±0.18  390±41.8 | | | | | | | | | | 2.1±0.58  2.4±0.64 | 1.04±0.34  1.2±0.48 | 0.83±0.19  0.88±0.16 | 0.65±0.17  0.98±0.41 |
| c4-OH-Pro | LB  LSL | 0.06±0.02  0.09±0.02 | | | | | | | | | | 0.03±0.03  0.02±0.01 | -  - | -  - | -  - |
| Carnosine | LB  LSL | 8.34±1.28  11.1±2.53 | | | | | | | | | | 20.3±4.57  26.7±3.8 | 7.5±1.02  10.22±2.71 | 9.20±2.31  9.11±1.84 | 10.67±1.65  10.69±1.63 |
| Creatinine | LB  LSL | 1.04±0.52  1.04±0.42 | | | | | | | | | | 2.94±0.63  2.95±0.54 | 3.08±0.73  2.34±0.82 | 2.21±0.42  1.76±0.41 | 3.02±0.33  2.44±0.50 |
| DOPA | LB  LSL | 0.09±0.02  0.08±0.01 | | | | | | | | | | 0.05±0.01  0.05±0.01 | 0.07±0.01  0.07±0.001 | 0.04±0.01  0.05±0.001 | 0.11±0.01  0.1±0.01 |
| Dopamine | LB  LSL | 0.23±0.12  0.19±0.03 | | | | | | | | | | 0.25±0.22  0.13±0.03 | -  0.24±0.14 | 0.26±0.20  - | 0.26±0.11  0.20±0.09 |
| Histamine | LB  LSL | 0.02±0.01  0.03±0.03 | | | | | | | | | | 0.15±0.06  0.16±0.03 | 0.24±0.44  0.14±0.10 | 0.11±0.04  0.17±0.24 | 0.18±0.02  0.19±0.05 |
| Kynurenine | LB  LSL | 0.18±0.05  0.12±0.04 | | | | | | | | | | 0.57±0.25  0.44±0.24 | 0.36±0.22  0.18±0.08 | 0.51±0.19  0.16±0.03 | 0.37±0.15  0.26±0.07 |
| Met-SO | LB  LSL | 12.90±2.54  14.30±2.16 | | | | | | | | | | 18.70±3.23  19.9±3.38 | 15.50±3.21  13.47±4.17 | 13.14±1.26  11.72±2.72 | 10.01±1.65  9.03±1.32 |
| Nitro-Tyr | LB  LSL | -  - | | | | | | | | | | -  - | -  - | -  - | -  - |
| PEA | LB  LSL | -  - | | | | | | | | | | -  - | -  - | -  - | -  - |
| Putrescine | LB  LSL | 0.76±0.24  1.1±0.35 | | | | | | | | | | 0.91±0.47  0.96±0.32 | 2.02±1.91  1.69±0.69 | 1.10±0.56  0.95±0.77 | 1.26±0.47  1.06±0.33 |
| Sarcosine | LB  LSL | 22.5±3.72  23.1±5.76 | | | | | | | | | | 21.6±2.80  24±4.12 | 12±5.00  7.9±2.34 | 10.86±2.86  9.96±3.24 | 10.1±2.50  8.57±1.45 |
| SDMA | LB  LSL | 0.45±0.03  0.42±0.05 | | | | | | | | | | 0.62±0.07  0.53±0.11 | 0.57±0.1  0.47±0.05 | 0.41±0.06  0.36±0.07 | 0.35±0.04  0.31±0.11 |
| Serotonin | LB  LSL | 3.46±5.61  3.96±5.54 | | | | | | | | | | 2.83±8.49  8.60±10.4 | 0.78±2.05  12.21±19.6 | 11.32±22.6  1.50±3.07 | 11.3±15.3  10.6±25.1 |
| Spermidine | LB  LSL | 0.25±0.05  0.26±0.05 | | | | | | | | | | 0.5±0.40  0.43±0.12 | 0.34±0.18  0.36±0.13 | 0.33±0.11  0.35±0.37 | 0.73±0.36  0.5±0.31 |
| Spermine | LB  LSL | 0.33±0.06  0.34±0.05 | | | | | | | | | | 0.58±0.21  0.63±0.18 | 0.3±0.06  0.36±0.13 | 0.34±0.05  0.35±0.06 | 0.78±0.36  0.72±0.40 |
| t4-OH-Pro | LB  LSL | 118.9±19.6  150.5±21.05 | | | | | | | | | | 134.4±25.0  134±20.3 | 30.8±11.4  25.3±5.66 | 20.5±4.97  18.8±4.54 | 18.3±3.90  19.2±2.90 |
| Taurine | LB  LSL | 150.4±45.7  235.1±125 | | | | | | | | | | 423.8±274  774.5±368 | 337.2±121  380±154 | 242.3±123  326.4±95.8 | 220.7±69.5  346.9±146 |
| **Metabolite**  (µmol/l) | **Strain** | **Week 10** | | | | | | | | | | **Week 16** | **Week 24** | **Week 30** | **Week 60** |
| **Carnitine** |  |  |  |  |  |  |  |  |  |  |  |  |  |  |  |
| C0 | LB  LSL | 8.93±1.74  8.74±1.00 | | | | | | | | | | 15±2.98  16.7±3.90 | 17.7±5.72  14.47±3.11 | 16.4 ±2.46  12.5±1.84 | 18.9±3.84  13.1±3.33 |
| **Acylcarnitines** |  |  | | | | | | | | | |  |  |  |  |
| C2 | LB  LSL | 2.25±0.49  2.37±0.31 | | | | | | | | | | 3.25±0.89  3.68±1.03 | 4.67±1.13  4.1±1.10 | 4.44±0.76  4.23±0.71 | 4.94±1.38  4.28±1.54 |
| C3 | LB  LSL | 0.31±0.08  0.39±0.08 | | | | | | | | | | 0.37±0.16  0.41±0.19 | 0.8±0.46  0.68±0.13 | 0.58±0.11  0.48±0.17 | 1.53±0.76  1.1±0.72 |
| C3-DC (C4-OH) | LB | 0.11±0.03 | | | | | | | | | | 0.08±0.01 | 0.05±0.01 | 0.05±0.01 | 0.05±0.004 |
|  | LSL | 0.11±0.01 | | | | | | | | | | 0.09±20.6 | 0.06±0.01 | 0.06±0.02 | 0.05±0.003 |
| C3-OH | LB  LSL | 0.01±0.002  0.01±0.002 | | | | | | | | | | 0.05±0.01  0.05±0.005 | 0.05±0.01  0.05±0.01 | 0.05±0.01  0.05±0.01 | 0.04±0.005  0.04±0.008 |
| C3:1 | LB  LSL | 0.01±0.001  0.01±0.001 | | | | | | | | | | 0.03±0.004  0.03±0.01 | 0.03±0.01  0.03±0.01 | 0.03±0.01  0.02±0.005 | 0.04±0.01  0.03±0.005 |
| C4 | LB | 0.08±0.01 | | | | | | | | | | 0.11±0.02 | 0.15±0.04 | 0.14±0.01 | 0.16±0.05 |
| C4:1 | LSL  LB | 0.08±0.01  0.04±0.003 | | | | | | | | | | 0.11±0.02  0.05±0.01 | 0.11±0.01  0.05±0.01 | 0.1±0.03  0.04±0.01 | 0.12±0.02  0.05±0.01 |
| C5 | LSL  LB | 0.03±0.004  0.05±0.01 | | | | | | | | | | 0.05±0.01  0.11±0.02 | 0.05±0.01  0.13±0.03 | 0.04±0.01  0.13±0.02 | 0.05±0.006  0.14±0.04 |
| C5-DC (C6-OH) | LSL  LB | 0.06±0.01  0.01±0.003 | | | | | | | | | | 0.13±0.04  0.03±0.01 | 0.11±0.02  0.02±0.01 | 0.1±0.02  0.02±0.01 | 0.1±0.01  0.03±0.004 |
| C5-M-DC | LSL  LB | 0.02±0.001  0.03±0.003 | | | | | | | | | | 0.03±0.01  0.03±0.01 | 0.03±0.01  0.05±0.01 | 0.03±0.01  0.04±0.01 | 0.03±0.006  0.04±0.01 |
| C5-OH (C3-DC-M) | LSL  LB | 0.03±0.003  0.03±0.01 | | | | | | | | | | 0.03±0.01  0.05±0.004 | 0.05±0.01  0.07±0.02 | 0.03±0.01  0.06±0.01 | 0.03±0.004  0.07±0.02 |
| C5:1 | LSL  LB | 0.03±0.01  0.01±0.002 | | | | | | | | | | 0.06±0.01  0.02±0.01 | 0.06±0.01  0.02±0.01 | 0.06±0.01  0.02±0.01 | 0.07±0.01  0.03±0.003 |
| C5:1-DC | LSL  LB | 0.01±0.002  0.01±0.001 | | | | | | | | | | 0.02±0.01  0.02±0.01 | 0.02±0.004  0.02±0.003 | 0.03±0.01  0.02±0.01 | 0.03±0.005  0.02±0.005 |
| C6 (C4:1-DC) | LSL  LB | 0.01±0.002  0.03±0.003 | | | | | | | | | | 0.02±0.002  0.04±0.01 | 0.02±0.003  0.04±0.01 | 0.02±0.01  0.04±0.01 | 0.018±0.002  0.05±0.01 |
| C6:1 | LSL  LB | 0.03±0.004  0.02±0.002 | | | | | | | | | | 0.04±0.005  0.02±0.003 | 0.04±0.01  0.02±0.002 | 0.04±0.01  0.02±0.004 | 0.05±0.005  0.03±0.003 |
| C7-DC | LSL  LB | 0.02±0.001  0.01±0.001 | | | | | | | | | | 0.02±0.01  0.02±0.004 | 0.02±0.003  0.01±0.003 | 0.02±0.01  0.01±0.01 | 0.03±0.007  0.02±0.002 |
| C8 | LSL  LB | 0.01±0.002  0.06±0.01 | | | | | | | | | | 0.02±0.003  0.05±0.01 | 0.01±0.004  0.06±0.01 | 0.01±0.01  0.06±0.01 | 0.02±0.004  0.06±0.01 |
| C9 | LSL  LB | 0.07±0.01  0.01±0.001 | | | | | | | | | | 0.06±0.01  0.02±0.002 | 0.06±0.01  0.02±0.004 | 0.06±0.01  0.02±0.004 | 0.06±0.008  0.03±0.003 |
| C10 | LSL  LB | 0.01±0.002  0.07±0.01 | | | | | | | | | | 0.02±0.004  0.08±0.01 | 0.02±0.01  0.07±0.01 | 0.02±0.004  0.07±0.01 | 0.03±0.006  0.09±0.01 |
| C10:1 | LSL  LB | 0.07±0.01  0.09±0.01 | | | | | | | | | | 0.08±0.01  0.07±0.01 | 0.07±0.01  0.07±0.01 | 0.07±0.01  0.07±0.01 | 0.09±0.01  0.12±0.02 |
| C10:2 | LSL  LB | 0.08±0.01  0.03±0.002 | | | | | | | | | | 0.06±0.01  0.05±0.01 | 0.07±0.01  0.05±0.01 | 0.07±0.01  0.04±0.003 | 0.12±0.01  0.05±0.01 |
| **Metabolite**  (µmol/l) | **Strain** | **Week 10** | | | | | | | | | | **Week 16** | **Week 24** | **Week 30** | **Week 60** |
| C12 | LSL  LB | 0.03±0.002  0.03±0.001 | | | | | | | | | | 0.04±0.01  0.03±0.004 | 0.05±0.01  0.05±0.01 | 0.04±0.01  0.05±0.01 | 0.05±0.005  0.04±0.01 |
| C12-DC | LSL  LB | 0.03±0.003  0.08±0.01 | | | | | | | | | | 0.04±0.004  0.11±0.01 | 0.04±0.01  0.08±0.01 | 0.05±0.01  0.09±0.01 | 0.04±0.01  0.07±0.01 |
| C12:1 | LSL  LB | 0.08±0.01  0.05±0.003 | | | | | | | | | | 0.11±0.01  0.03±0 | 0.10±0.01  0.03±0.01 | 0.09±0.01  0.04±0.003 | 0.07±0.004  0.05±0.01 |
| C14 | LSL  LB | 0.04±0.004  0.02±0.003 | | | | | | | | | | 0.04±0.02  0.02±0.01 | 0.04±0.01  0.03±0.01 | 0.04±0.01  0.03±0.01 | 0.05±0.004  0.02±0.004 |
| C14:1 | LSL  LB | 0.02±0.002  0.03±0.004 | | | | | | | | | | 0.02±0.003  0.04±0.01 | 0.03±0.01  0.03±0.005 | 0.03±0.01  0.02±0.004 | 0.03±0.003  0.02±0.01 |
| C14:1-OH | LSL  LB | 0.03±0.004  0.01±0.002 | | | | | | | | | | 0.05±0.01  0.01±0.001 | 0.03±0.01  0.01±0.001 | 0.04±0.01  0.01±0.004 | 0.02±0.007  0.01±0.005 |
| C14:2 | LSL  LB | 0.01±0.002  0.005±0.001 | | | | | | | | | | 0.01±0.003  0.01±0.002 | 0.01±0.003  0.01±0.002 | 0.01±0.003  0.01±0.01 | 0.01±0.003  0.02±0.01 |
| C14:2-OH | LSL  LB | 0.005±0.001  0.01±0.001 | | | | | | | | | | 0.01±0.002  0.01±0.001 | 0.02±0.01  0.01±0.002 | 0.01±0.003  0.01±0.004 | 0.01±0.001  0.02±0.004 |
| C16 | LSL  LB | 0.01±0.001  0.02±0.005 | | | | | | | | | | 0.01±0.001  0.03±0.01 | 0.02±0.004  0.02±0.01 | 0.01±0.002  0.02±0.01 | 0.02±0.003  0.02±0.01 |
| C16-OH | LSL  LB | 0.02±0.003  0.01±0.001 | | | | | | | | | | 0.02±0.01  0.01±0.002 | 0.03±0.004  0.01±0.003 | 0.03±0.01  0.01±0.003 | 0.03±0.009  0.02±0.01 |
| C16:1 | LSL  LB | 0.01±0.001  0.01±0.001 | | | | | | | | | | 0.01±0.003  0.01±0.003 | 0.01±0.003  0.02±0.01 | 0.01±0.003  0.02±0.004 | 0.02±0.004  0.1±0.01 |
| C16:1-OH | LSL  LB | 0.01±0.001  0.005±0.001 | | | | | | | | | | 0.01±0.002  0.01±0.001 | 0.02±0.004  0.01±0.001 | 0.02±0.01  0.01±0.003 | 0.1±0.01  0.02±0.004 |
| C16:2 | LSL  LB | 0.004±0.001  0.003±0.001 | | | | | | | | | | 0.01±0.003  0.01±0.001 | 0.01±0.004  0.01±0.005 | 0.01±0.01  0.02±0.003 | 0.02±0.001  0.01±0.004 |
| C16:2-OH | LSL  LB | 0.003±0.001  0.01±0.001 | | | | | | | | | | 0.01±0.002  0.01±0.003 | 0.02±0.004  0.01±0.004 | 0.02±0.004  0.01±0.004 | 0.01±0.002  0.02±0.005 |
| C18 | LSL  LB | 0.01±0.001  0.01±0.004 | | | | | | | | | | 0.01±0.004  0.01±0.01 | 0.01±0.002  0.02±0.004 | 0.01±0.001  0.02±0.005 | 0.02±0.003  0.02±0.01 |
| C18:1 | LSL  LB | 0.01±0.001  0.03±0.01 | | | | | | | | | | 0.01±0.01  0.04±0.01 | 0.02±0.01  0.04±0.02 | 0.02±0.02  0.04±0.01 | 0.02±0.01  0.03±0.01 |
| C18:1-OH | LSL  LB | 0.02±0.003  0.01±0.003 | | | | | | | | | | 0.03±0.01  0.02±0.004 | 0.04±0.01  0.02±0.01 | 0.05±0.02  0.02±0.01 | 0.03±0.01  0.05±0.01 |
| C18:2 | LSL  LB  LSL | 0.01±0.002  0.01±0.003  0.01±0.001 | | | | | | | | | | 0.02±0.002  0.01±0.004  0.01±0.002 | 0.02±0.01  0.01±0.005  0.02±0.004 | 0.02±0.01  0.01±0.001  0.02±0.01 | 0.05±0.01  0.01±0.005  0.01±0.005 |
| **PCs** |  |  | | | | | | | | | |  |  |  |  |
| PC aa C24:0 | LB  LSL | 0.19±0.02  0.17±0.19 | | | | | | | | | | 0.07±0.02  0.06±0.02 | 0.07±0.03  0.14±0.03 | 0.07±0.02  0.07±0.02 | 0.12±0.04  0.16±0.04 |
| PC aa C26:0 | LB  LSL | 0.63±0.20  0.6±0.20 | | | | | | | | | | 0.33±0.02  0.32±0.02 | 0.39±0.08  0.37±0.07 | 0.38±0.03  0.39±0.04 | 0.74±0.40  0.95±0.50 |
| PC aa C28:1 | LB  LSL | 0.26±0.08  0.24±0.14 | | | | | | | | | | 0.27±0.03  0.26±0.03 | 0.42±0.14  0.35±0.10 | 0.39±0.10  0.39±0.14 | 0.56±0.19  0.59±0.24 |
| **Metabolite**  (µmol/l) | **Strain** | **Week 10** | | | | | | | | | | **Week 16** | **Week 24** | **Week 30** | **Week 60** |
| PC aa C30:2 | LB  LSL | 0.16±0.15  0.14±0.17 | | | | | | | | | | 0.09±0.04  0.05±0.03 | 0.17±0.04  0.14±0.03 | 0.18±0.06  0.12±0.09 | 0.09±0.06  0.1±0.05 |
| PC aa C32:0 | LB  LSL | 24.3±5.96  28.5±4.20 | | | | | | | | | | 28.9±3.60  31.6±4.14 | 41.4±11.05  39.8±11.5 | 46.9±11.93  47±9.81 | 41.4±13.24  50.1±28.0 |
| PC aa C32:1 | LB  LSL | 8.09±3.94  8.56±2.95 | | | | | | | | | | 7.76±2.55  9.19±2.65 | 78.9±19.62  82.7±23.2 | 96.4±35.9  80±14.42 | 75.60±31.1  75±33.0 |
| PC aa C32:2 | LB  LSL | 1.5±0.41  1.47±0.19 | | | | | | | | | | 1.94±0.36  1.56±0.25 | 9.55±2.36  7.51±2.00 | 10.43±2.43  8.33±2.09 | 12.93±4.67  10.93±3.58 |
| PC aa C32:3 | LB  LSL | 0.46±0.06  0.25±0.03 | | | | | | | | | | 0.68±0.16  0.38±0.08 | 1.33±0.35  0.82±0.22 | 1.27±0.22  0.85±0.18 | 1.11±0.41  0.89±0.35 |
| PC aa C34:1 | LB  LSL | 155±43.0  177.4±37.9 | | | | | | | | | | 194.5±41.5  207.6±27.1 | 1180.5±348  1221.8±299 | 1303±226  1364.6±321 | 1055.2±242  1138.8±269 |
| PC aa C34:2 | LB  LSL | 231.2±49.0  258.4±27.9 | | | | | | | | | | 286±44.4  275±37.9 | 876.3±248  883.3±232 | 959.3±179.4  1013.5±247 | 949±232  1034.1±279 |
| PC aa C34:3 | LB  LSL | 11.25±3.23  13.2±1.62 | | | | | | | | | | 14.28±2.63  14±2.05 | 66.3±16.5  57.4±15.9 | 71.6±14.14  64.1±15.2 | 82.3±25.71  76.3±26.4 |
| PC aa C34:4 | LB  LSL | 0.8±0.13  0.74±0.12 | | | | | | | | | | 1.02±0.16  0.98±0.16 | 5.52±1.61  3.68±1.05 | 4.7±0.68  3.66±0.84 | 3.85±1.53  3.75±1.85 |
| PC aa C36:0 | LB  LSL | 1.48±0.23  2.12±0.40 | | | | | | | | | | 2.49±0.52  2.87±0.41 | 9.30±3.66  10.27±4.17 | 9.42±2.96  10.31±5.55 | 7.54±3.14  8.57±5.38 |
| PC aa C36:1 | LB  LSL | 69.9±18.6  100.7±23.8 | | | | | | | | | | 103±31.7  129.8±21.7 | 543.9±173  552.4±157 | 548.2±130  577.9±203 | 465.3±138  499.7±191 |
| PC aa C36:2 | LB  LSL | 239.7±47.5  297.7±37.2 | | | | | | | | | | 334.3±56.5  347.1±42.8 | 622.8±193  605.7±166 | 637.5±135  666.2±203 | 636.9±167  666±219 |
| PC aa C36:3 | LB  LSL | 65.7±16.9  80.6±11.8 | | | | | | | | | | 94.7±20.2  100.6±11.8 | 178.1±47.0  170.4±42.2 | 181.2±35.2  190.8±47.6 | 188.1±55.5  197.6±67.3 |
| PC aa C36:4 | LB  LSL | 107.5±16.1  126.6±19.1 | | | | | | | | | | 165.8±32.3  172.5±26.2 | 220.2±64.0  234.1±73.0 | 259.1±31.9  283.8±65.3 | 257.2±78.4  307.6.8±132 |
| PC aa C36:5 | LB  LSL | 8.25±2.17  10.74±1.63 | | | | | | | | | | 11.22±2.00  13.6±1.58 | 14.88±3.38  13.6±3.48 | 14.10±2.60  13.27±2.79 | 13.4±4.44  14.2±6.04 |
| PC aa C36:6 | LB  LSL | 0.3±0.04  0.33±0.06 | | | | | | | | | | 0.47±0.10  0.51±0.09 | 1.8±0.70  1.41±0.41 | 1.58±0.30  1.39±0.44 | 1.41±0.57  1.36±0.60 |
| PC aa C38:0 | LB  LSL | 0.73±0.08  0.89±0.12 | | | | | | | | | | 1.21±0.27  1.14±0.22 | 2.21±0.83  2.16±0.76 | 2.46±0.53  2.29±0.85 | 2.76±0.97  2.61±0.97 |
| PC aa C38:1 | LB  LSL | 0.52±0.1  0.80±0.21 | | | | | | | | | | 1.39±0.52  1.63±0.25 | 2.85±0.77  3.43±1.15 | 2.45±0.46  2.97±0.96 | 0.82±0.39  1.19±0.99 |
| PC aa C38:4 | LB  LSL | 142.6±24.2  176.1±30.1 | | | | | | | | | | 267.1±66.6  271.6±40.7 | 263±112.5  266.6±89.7 | 279.7±79.6  312.1±134 | 261.8±81.3  291.4±130 |
| PC aa C38:5 | LB  LSL | 32.1±5.74  38±6.85 | | | | | | | | | | 51.2±10.8  55.1±7.39 | 133.5±42.7  113.2±30.1 | 143.2±36.1  116.7±34.1 | 128.7±44.0  114.2±66.2 |
| PC aa C38:6 | LB  LSL | 25.2±3.19  34±5.44 | | | | | | | | | | 49.8±12.6  52.7±9.84 | 170±75.1  165.2±51.8 | 178.7±44.3  175.2±67.0 | 199.1±68.4  190.6±80.6 |
| PC aa C40:1 | LB  LSL | 0.24±0.03  0.26±0.04 | | | | | | | | | | 0.30±0.04  0.31±0.02 | 0.49±0.13  0.46±0.14 | 0.47±0.06  0.5±0.09 | 0.46±0.11  0.53±0.18 |
| PC aa C40:2 | LB  LSL | 0.36±0.05  0.38±0.04 | | | | | | | | | | 0.56±0.11  0.53±0.07 | 0.51±0.15  0.44±0.13 | 0.48±0.11  0.47±0.11 | 0.43±0.12  0.47±0.17 |
| **Metabolite**  (µmol/l) | **Strain** | **Week 10** | | | | | | | | | | **Week 16** | **Week 24** | **Week 30** | **Week 60** |
| PC aa C40:3 | LB  LSL | 0.71±0.09  0.92±0.14 | | | | | | | | | | 1.13±0.39  1.35±0.16 | 0.88±0.32  0.96±0.26 | 0.83±0.25  0.87±0.17 | 0.66±0.19  0.87±0.65 |
| PC aa C40:5 | LB  LSL | 12.41±2.40  15±3.30 | | | | | | | | | | 18.4±4.9  19.1±2.81 | 50.1±17.6  44.3±14.0 | 48.7±15.1  42.5±14.8 | 39.7±12.9  40.2±29.1 |
| PC aa C40:6 | LB  LSL | 15.8±2.53  23.2±4.05 | | | | | | | | | | 33.5±9.25  34.7±5.64 | 69.6±28.1  68.5±23.2 | 67.5±18.5  70.4±32.3 | 70.8±24.0  72.7±39.9 |
| PC aa C42:0 | LB  LSL | 0.05±0.01  0.06±0.02 | | | | | | | | | | 0.09±0.02  0.07±0.02 | 0.14±0.05  0.13±0.04 | 0.11±0.02  0.14±0.05 | 0.13±0.02  0.12±0.05 |
| PC aa C42:1 | LB  LSL | 0.07±0.01  0.08±0.01 | | | | | | | | | | 0.12±0.03  0.12±0.01 | 0.19±0.06  0.16±0.05 | 0.17±0.04  0.15±0.03 | 0.14±0.05  0.15±0.03 |
| PC aa C42:2 | LB  LSL | 0.16±0.02  0.15±0.02 | | | | | | | | | | 0.24±0.03  0.22±0.02 | 0.24±0.07  0.27±0.09 | 0.23±0.03  0.24±0.03 | 0.25±0.06  0.27±0.09 |
| PC aa C42:4 | LB  LSL | 0.48±0.08  0.54±0.09 | | | | | | | | | | 0.65±0.15  0.63±0.14 | 0.75±0.28  0.81±0.22 | 0.78±0.21  0.81±0.33 | 0.97±0.26  1.18±0.89 |
| PC aa C42:5 | LB  LSL | 0.39±0.05  0.48±0.08 | | | | | | | | | | 0.32±0.15  0.65±0.14 | 1.07±0.34  1±0.29.0 | 1.04±0.22  0.96±0.28 | 1.12±0.31  1.16±0.78 |
| PC aa C42:6 | LB  LSL | 0.35±0.05  0.38±0.05 | | | | | | | | | | 0.63±0.15  0.63±0.09 | 1.3±0.40  1.1±0.25 | 1.14±0.27  0.94±0.29 | 1.17±0.32  1.06±0.50 |
| PC ae C30:0 | LB  LSL | 0.1±0.01  0.1±0.01 | | | | | | | | | | 0.16±0.02  0.14±0.02 | 0.16±0.03  0.15±0.04 | 0.16±0.03  0.14±0.03 | 0.16±0.04  0.15±0.04 |
| PC ae C30:1 | LB  LSL | 0.04±0.03  0.04±0.05 | | | | | | | | | | 0.04±0.03  0.03±0.02 | 0.06±0.04  0.07±0.04 | 0.04±0.02  0.07±0.05 | 0.1±0.08  0.14±0.11 |
| PC ae C30:2 | LB  LSL | 0.11±0.03  0.08±0.02 | | | | | | | | | | 0.05±0.001  0.05±0.01 | 0.06±0.02  0.05±0.01 | 0.05±0.01  0.06±0.02 | 0.09±0.03  0.11±0.05 |
| PC ae C32:1 | LB  LSL | 1.42±0.13  1.43±0.28 | | | | | | | | | | 1.99±0.30  1.89±0.37 | 1.88±0.52  1.53±0.40 | 1.85±0.42  1.55±0.50 | 1.67±0.46  1.58±0.68 |
| PC ae C32:2 | LB  LSL | 0.18±0.02  0.17±0.05 | | | | | | | | | | 0.27±0.05  0.26±0.05 | 0.32±0.09  0.27±0.06 | 0.34±0.08  0.28±0.08 | 0.3±0.08  0.29±0.10 |
| PC ae C34:0 | LB  LSL | 0.87±0.11  0.93±0.08 | | | | | | | | | | 1.27±0.21  1.21±0.25 | 1.11±0.38  0.98±0.24 | 1.03±0.22  1.09±0.31 | 0.96±0.29  1.17±0.66 |
| PC ae C34:1 | LB  LSL | 5.31±0.61  5.54±0.82 | | | | | | | | | | 7.79±1.22  6.93±1.31 | 12.79±4.21  11.46±3.31 | 14.5±2.37  12.92±4.03 | 13.15±4.02  13.47±4.54 |
| PC ae C34:2 | LB  LSL | 6.9±1.07  7.72±1.11 | | | | | | | | | | 10.7±1.88  10.14±1.66 | 14.91±5.07  12.98±3.43 | 16.1±2.92  14.73±4.41 | 15.97±4.87  16±5.37 |
| PC ae C34:3 | LB  LSL | 1.17±0.16  1.37±0.22 | | | | | | | | | | 1.38±0.23  1.31±0.20 | 1.83±0.42  1.76±0.41 | 1.73±0.30  1.74±0.42 | 1.95±0.59  2.14±0.74 |
| PC ae C36:0 | LB  LSL | 0.34±0.05  1.34±0.04 | | | | | | | | | | 0.48±0.07  0.49±0.09 | 1.21±0.40  0.93±0.25 | 1.2±0.21  0.97±0.24 | 1.08±0.30  1.16±0.67 |
| PC ae C36:1 | LB  LSL | 1.96±0.22  2.29±0.32 | | | | | | | | | | 2.71±0.45  2.66±0.39 | 14.21±5.24  12.90±3.91 | 15.4±2.5  15.90±5.63 | 15.38±4.82  16.07±5.28 |
| PC ae C36:2 | LB  LSL | 4.51±0.70  5.05±0.59 | | | | | | | | | | 6.2±0.88  5.66±0.89 | 16.24±6.05  14.5±5.02 | 16.8±2.62  17.7±6.17 | 18.7±5.74  20.7±5.64 |
| PC ae C36:3 | LB  LSL | 2.58±0.32  2.73±0.39 | | | | | | | | | | 3.31±0.52  3.23±0.49 | 6.58±2.02  5.62±1.56 | 6.15±1.01  6.01±2.11 | 6.65±1.99  6.87±2.06 |
| PC ae C36:4 | LB  LSL | 15.7±1.64  14±2.36 | | | | | | | | | | 19.8±3.29  17±3.46 | 7.21±2.03  5.96±1.64 | 6.7±1.41  6.19±2.08 | 6.16±1.80  6.52±4.20 |
| **Metabolite**  (µmol/l) | **Strain** | **Week 10** | | | | | | | | | | **Week 16** | **Week 24** | **Week 30** | **Week 60** |
| PC ae C36:5 | LB  LSL | 2.54±0.27  2.63±0.48 | | | | | | | | | | 3.23±0.58  3.06±0.51 | 1.51±0.44  1.51±0.41 | 1.50±0.26  1.61±0.48 | 1.54±0.45  1.79±0.97 |
| PC ae C38:0 | LB  LSL | 0.91±0.15  1.08±0.20 | | | | | | | | | | 1.60±0.44  1.71±0.30 | 4.26±1.67  3.38±0.91 | 3.49±0.76  3.05±1.23 | 3.28±1.23  2.8±1.39 |
| PC ae C38:1 | LB  LSL | 0.7±0.15  0.9±0.16 | | | | | | | | | | 1.03±0.30  1.05±0.15 | 4.13±1.35  3.50±0.88 | 3.61±0.66  3.41±1.32 | 3.57±1.06  3.6±1.79 |
| PC ae C38:2 | LB  LSL | 1.70±0.49  2.08±0.35 | | | | | | | | | | 2.79±0.52  2.63±0.43 | 4.44±1.47  3.78±1.06 | 4.2±0.86  4.21±1.38 | 3.85±1.11  4.02±1.67 |
| PC ae C38:3 | LB  LSL | 1.08±0.18  1.31±0.18 | | | | | | | | | | 1.71±0.38  1.74±0.23 | 2.04±0.6  1.7±0.42 | 1.76±0.31  1.67±0.57 | 1.56±0.44  1.63±0.67 |
| PC ae C38:4 | LB  LSL | 10.79±1.22  11.18±1.48 | | | | | | | | | | 15.31±2.96  14.2±2.94 | 6.99±2.08  6.47±1.79 | 6.86±1.26  6.98±2.13 | 6.46±1.69  7.57±4.24 |
| PC ae C38:5 | LB  LSL | 8.02±1.00  7.53±1.23 | | | | | | | | | | 10.95±1.92  9.82±2.02 | 5.72±1.54  4.98±1.45 | 5.90±1.05  5.11±1.45 | 5.4±1.47  5.5±2.82 |
| PC ae C38:6 | LB  LSL | 1.98±0.20  2.09±0.38 | | | | | | | | | | 2.80±0.61  2.53±0.61 | 2.74±1.12  2.70±1.06 | 3.09±0.50  3.06±0.91 | 3.59±1.00  4.1±1.03 |
| PC ae C40:1 | LB  LSL | 0.47±0.09  0.58±0.13 | | | | | | | | | | 1.06±0.33  1.20±0.16 | 0.89±0.40  0.81±0.21 | 0.67±0.15  0.67±0.24 | 0.64±0.27  0.67±0.38 |
| PC ae C40:2 | LB  LSL | 0.35±0.05  0.38±0.04 | | | | | | | | | | 0.49±0.06  0.49±0.06 | 0.53±0.15  0.53±0.14 | 0.43±0.04  0.43±0.12 | 0.33±0.12  0.44±0.23 |
| PC ae C40:3 | LB  LSL | 0.38±0.05  0.43±0.07 | | | | | | | | | | 0.53±0.11  0.58±0.07 | 0.44±0.14  0.44±0.13 | 0.4±0.07  0.47±0.14 | 0.37±0.10  0.4±0.13 |
| PC ae C40:4 | LB  LSL | 2.2±0.31  2.7±0.39 | | | | | | | | | | 3.4±0.66  3.38±0.56 | 3.17±1.16  3.26±1.17 | 3.44±0.88  3.79±1.26 | 3.89±1.21  4.5±2.08 |
| PC ae C40:5 | LB  LSL | 2.11±0.31  2.31±0.33 | | | | | | | | | | 3.05±0.61  2.96±0.51 | 3.8±1.34  3.56±1.30 | 4.27±0.98  4.09±1.38 | 4.7±1.61  4.45±2.02 |
| PC ae C40:6 | LB  LSL | 1.42±0.19  1.68±0.27 | | | | | | | | | | 2.36±0.55  2.21±0.45 | 4.2±2.03  4.11±1.76 | 4.69±1.24  5.04±2.38 | 5.32±2.02  6.02±1.71 |
| PC ae C42:0 | LB  LSL | 0.59±0.04  0.63±0.08 | | | | | | | | | | 0.66±0.13  0.63±0.06 | 1.23±0.30  1.01±0.20 | 1.07±0.23  0.94±0.23 | 1.2±0.26  0.98±0.24 |
| PC ae C42:1 | LB  LSL | 0.32±0.07  0.36±0.10 | | | | | | | | | | 0.72±0.16  0.7±0.06 | 0.84±0.32  0.67±0.17 | 0.66±0.14  0.57±0.21 | 0.78±0.29  0.67±0.16 |
| PC ae C42:2 | LB  LSL | 0.23±0.04  0.26±0.06 | | | | | | | | | | 0.42±0.12  0.46±0.03 | 0.26±0.11  0.23±0.05 | 0.22±0.06  0.21±0.08 | 0.19±0.07  0.22±0.11 |
| PC ae C42:3 | LB  LSL | 0.17±0.03  0.23±0.06 | | | | | | | | | | 0.38±0.13  0.43±0.05 | 0.18±0.07  0.21±0.08 | 0.15±0.05  0.19±0.06 | 0.15±0.07  0.19±0.11 |
| PC ae C42:4 | LB  LSL | 0.11±0.03  0.13±0.02 | | | | | | | | | | 0.2±0.05  0.17±0.03 | 0.3±0.14  0.31±0.14 | 0.34±0.09  0.39±0.18 | 0.41±0.13  0.51±0.27 |
| PC ae C42:5 | LB  LSL | 0.49±0.04  0.51±0.03 | | | | | | | | | | 0.53±0.07  0.5±0.05 | 0.74±0.21  0.67±0.13 | 0.67±0.11  0.68±019 | 0.79±0.17  0.81±0.29 |
| PC ae C44:3 | LB  LSL | 0.05±0.01  0.06±0.02 | | | | | | | | | | 0.1±0.02  0.1±0.02 | 0.13±0.04  0.1±0.03 | 0.12±0.09  0.09±0.04 | 0.14±0.06  0.11±0.04 |
| PC ae C44:4 | LB  LSL | 0.06±0.01  0.08±0.02 | | | | | | | | | | 0.11±0.03  0.1±0.03 | 0.11±0.04  0.09±0.05 | 0.09±0.04  0.1±0.06 | 0.09±0.04  0.11±0.07 |
| PC ae C44:5 | LB  LSL | 0.06±0.01  0.08±0.01 | | | | | | | | | | 0.08±0.01  0.09±0.001 | 0.2±0.11  0.23±0.09 | 0.24±0.11  0.27±0.10 | 0.27±0.10  0.29±0.09 |
| **Metabolite**  (µmol/l) | **Strain** | **Week 10** | | | | | | | | | | **Week 16** | **Week 24** | **Week 30** | **Week 60** |
| PC ae C44:6 | LB  LSL | 0.06±0.01  0.08±0.01 | | | | | | | | | | 0.1±0.02  0.09±0.01 | 0.22±0.10  0.18±0.06 | 0.23±0.05  0.26±0.07 | 0.24±0.07  0.26±0.10 |
| **LysoPCs** |  |  | | | | | | | | | |  |  |  |  |
| LysoPC a C14:0 | LB  LSL | 4.02±0.25  3.91±0.13 | | | | | | | | | | 4.61±0.32  4.52±0.29 | 3.13±0.21  3.12±0.27 | 2.91±0.17  2.83±0.22 | 4.17±0.33  4.06±0.15 |
| LysoPC a C16:0 | LB  LSL | 11.58±2.14  11.42±1.86 | | | | | | | | | | 20.30±4.37  22.6±6.17 | 6.36±1.83  6.73±1.25 | 7.91±1.80  8.42±0.96 | 12.11±3.00  13.2±4.32 |
| LysoPC a C16:1 | LB  LSL | 0.41±0.07  0.34±0.08 | | | | | | | | | | 0.69±0.23  0.63±0.15 | 0.52±0.11  0.42±0.14 | 0.50±1.12  0.49±0.08 | 0.67±0.18  0.57±0.14 |
| LysoPC a C17:0 | LB  LSL | 0.13±0.04  0.11±0.03 | | | | | | | | | | 0.31±0.08  0.31±0.08 | 0.14±0.01  0.11±0.05 | 0.12±0.02  0.12±0.04 | 0.16±0.03  0.12±0.03 |
| LysoPC a C18:0 | LB  LSL | 7.70±1.79  8.84±1.80 | | | | | | | | | | 14.93±3.8  18.1±4.15 | 2.22±0.69  2.4±0.65 | 2.44±0.55  2.6±0.56 | 3.43±0.70  3.67±1.43 |
| LysoPC a C18:1 | LB  LSL | 2.93±0.48  3.27±0.67 | | | | | | | | | | 4.59±1.17  4.91±0.78 | 2.96±0.63  3.03±0.58 | 3.27±0.63  3.36±0.58 | 5.41±1.28  5.88±1.72 |
| LysoPC a C18:2 | LB  LSL | 4.19±0.83  5.31±0.87 | | | | | | | | | | 5.85±0.86  5.91±0.87 | 2.24±0.43  2.22±0.45 | 2.15±0.31  2.29±0.37 | 4.02±1.25  4.73±1.24 |
| LysoPC a C20:3 | LB  LSL | 0.42±0.07  0.56±0.11 | | | | | | | | | | 0.57±0.18  0.74±0.13 | 1.25±0.37  1.42±0.42 | 1.34±0.26  1.5±0.28 | 0.91±0.19  1.01±0.23 |
| LysoPC a C20:4 | LB  LSL | 1.14±0.31  1.36±0.24 | | | | | | | | | | 2.16±0.58  2.40±0.58 | 0.61±0.12  0.74±0.22 | 0.69±0.16  0.76±0.14 | 0.8±0.16  1.03±0.38 |
| LysoPC a C24:0 | LB  LSL | 0.26±0.08  0.22±0.08 | | | | | | | | | | 0.22±0.07  0.19±0.09 | 0.22±0.10  0.16±0.07 | 0.17±0.08  0.16±0.05 | 0.21±0.06  0.21±0.05 |
| LysoPC a C26:0 | LB  LSL | 0.04±0.03  0.04±0.04 | | | | | | | | | | 0.14±0.04  0.14±0.05 | -  - | 0.15±0.04  0.16±0.06 | 0.31±0.11  0.42±0.27 |
| LysoPC a C26:1 | LB  LSL | 0.03±0.03  0.03±0.04 | | | | | | | | | | 0.1±0.02  0.17±0.01 | 0.13±0.03  0.12±0.01 | 0.11±0.02  - | 0.17±0.08  0.21±0.11 |
| LysoPC a C28:0 | LB  LSL | 0.08±0.04  0.09±0.07 | | | | | | | | | | 0.14±0.05  0.13±0.02 | -  - | 0.09±0.03  0.09±0.03 | 0.15±0.04  0.15±0.04 |
| LysoPC a C28:1 | LB  LSL | 0.08±0.08  0.06±0.08 | | | | | | | | | | 0.11±0.02  0.12±0.02 | 0.13±0.03  0.1±0.02 | 0.11±0.02  0.11±0.02 | 0.12±0.04  0.15±0.05 |
| **SMs** |  |  |  |  |  |  |  |  |  |  |  |  |  |  |  |
| SM (OH) C14:1 | LB  LSL | 0.51±0.09  0.52±0.06 | | | | | | | | | | 0.77±0.11  0.67±0.12 | 0.48±0.14  0.44±0.11 | 0.5±0.06  0.46±0.12 | 0.47±0.13  0.52±0.18 |
| SM (OH) C16:1 | LB  LSL | 1.18±0.17  1.19±0.13 | | | | | | | | | | 1.92±0.37  1.66±0.36 | 0.79±0.24  0.72±0.16 | 0.76±0.12  0.75±0.27 | 0.77±0.18  0.9±0.32 |
| SM (OH) C22:1 | LB  LSL | 1.98±0.22  1.86±0.21 | | | | | | | | | | 2.64±0.39  2.43±0.47 | 0.83±0.14  0.9±0.20 | 0.89±0.16  0.92±0.24 | 0.79±0.15  0.93±0.46 |
| SM (OH) C22:2 | LB  LSL | 0.4±0.07  0.37±0.06 | | | | | | | | | | 0.48±0.07  0.4±0.08 | 0.24±0.05  0.23±0.06 | 0.23±0.06  0.23±0.08 | 0.25±0.08  0.26±0.10 |
| SM (OH) C24:1 | LB  LSL | 0.43±0.04  0.47±0.08 | | | | | | | | | | 0.55±0.1  0.56±0.1 | 0.2±0.04  0.25±0.07 | 0.2±0.06  0.27±0.06 | 0.16±0.04  0.24±0.13 |
| SM C16:0 | LB  LSL | 132.9±10.3  137.2±11.5 | | | | | | | | | | 175±26.2  181.5±30 | 134.9±38.8  135.5±32.6 | 136.3±25.0  139.7±40.0 | 140.6±31.6  159.7±55.1 |
| SM C16:1 | LB  LSL | 0.62±0.05  0.58±0.08 | | | | | | | | | | 0.76±0.14  0.77±0.11 | 0.84±0.26  0.73±0.16 | 0.83±0.14  0.77±0.25 | 0.86±0.20  0.83±0.32 |
| **Metabolite**  (µmol/l) | **Strain** | **Week 10** | | | | | | | | | | **Week 16** | **Week 24** | **Week 30** | **Week 60** |
| SM C18:0 | LB  LSL | 14.5±1.43  17.6±2.32 | | | | | | | | | | 23.2±4.66  25.9±4.51 | 8.24±2.41  9.16±2.43 | 8.9±1.60  10.6±2.89 | 9.59±1.91  11.4±4.21 |
| SM C18:1 | LB  LSL | 0.76±0.14  0.65±0.12 | | | | | | | | | | 1.24±0.27  1.07±0.14 | 0.78±0.24  0.74±0.23 | 0.94±0.29  0.97±0.38 | 1.15±0.32  1.03±0.32 |
| SM C20:2 | LB  LSL | 0.07±0.03  0.11±0.04 | | | | | | | | | | 0.11±0.05  0.12±0.05 | 0.09±0.10  0.06±0.06 | 0.1±0.07  0.07±0.04 | 0.2±0.09  0.19±0.11 |
| SM C22:3 | LB  LSL | -  - | | | | | | | | | | -  - | -  - | -  - | -  - |
| SM C24:0 | LB  LSL | 8.13±1.18  9.74±1.32 | | | | | | | | | | 11.32±1.90  12.48±1.75 | 6.8±1.57  6.37±1.08 | 5.69±0.83  5.8±1.55 | 5.34±1.19  5.44±2.10 |
| SM C24:1 | LB  LSL | 13.9±1.29  14.5±2.17 | | | | | | | | | | 19.2±3.46  18.9±2.28 | 6.24±1.30  5.98±1.68 | 5.27±0.91  5.77±1.48 | 5.47±1.06  6.23±5.33 |
| SM C26:0 | LB  LSL | 0.03±0.01  0.03±0.02 | | | | | | | | | | 0.04±0.02  0.05±0.02 | 0.04±0.03  0.04±0.02 | 0.03±0.02  0.03±0.02 | 0.02±0.01  0.04±0.02 |
| SM C26:1 | LB  LSL | 0.03±0.01  0.03±0.01 | | | | | | | | | | 0.09±0.04  0.11±0.05 | 0.03±0.02  0.04±0.02 | 0.05±0.03  0.06±0.05 | 0.03±0.02  0.04±0.05 |
| **Hexoses** |  |  | | | | | | | | | |  |  |  |  |
| Sum of hexoses | LB  LSL | 17245±1334  16482±1541 | | | | | | | | | | 22439±3172  21253±3158 | 19324±3507  17995±2711 | 16293±1879  15414±1587 | 14896±2862  13257±2138 |

Metabolites concentration of LB and LSL hens at 10, 16, 24, 30 and 60 weeks of age (n = 10). Values are showed as means ± SD. Concentration of metabolites that could not be measured are indicated as “-“. Abbreviations: ADMA (Asymmetric dimethylarginine), alpha-AAA (alpha-Aminoadipic acid), c4-OH-Pro (cis-4-Hydroxyproline), DOPA (Dihydroxyphenylalanine), Met-SO (Methionine-Sulfoxide), Nitro-Tyr (Nitrotyrosine), PEA (Phenylethylamine), SDMA (Symmetric dimethylarginine), t4-OH-Pro (trans-4-Hydroxyproline), C0 (free Carnitine), C2 (Acetylcarnitine), C3 (Propionylcarnitine), C3:1 (Propenoylcarnitine), C3-OH (Hydroxypropionylcarnitine), C4 (Butyrylcarnitine / Isobutyrylcarnitine), C4:1 (Butenoylcarnitine), C4-OH (C3-DC) (Hydroxybutyrylcarnitine (Malonylcarnitine)), C5 (Isovalerylcarnitine / 2-Methylbutyrylcarnitine / Valerylcarnitine), C5:1 (Tiglylcarnitine / 3-Methyl-crotonylcarnitine), C5:1-DC (Glutaconylcarnitine / Mesaconylcarnitine), C5-DC (C6-OH) (Glutarylcarnitine (Hydroxyhexanoylcarnitine [= Hydroxycaproylcarnitine])), C5-M-DC (Methylglutarylcarnitine), C5-OH (C3-DC-M) (Hydroxyisovalerylcarnitine / Hydroxy-2-methylbutyryl / Hydroxyvalerylcarnitine (Methylmalonylcarnitine)), C6 (C4:1-DC) (Hexanoylcarnitine [= Caproylcarnitine] (Fumarylcarnitine)), C6:1 (Hexenoylcarnitine), C7-DC (Pimelylcarnitine), C8 (Octanoylcarnitine [= Caprylylcarnitine]), C9 (Nonanoylcarnitine [= Pelargonylcarnitine]), C10 (Decanoylcarnitine [= Caprylcarnitine]), C10:1 (Decenoylcarnitine), C10:2 (Decadienoylcarnitine), C12 (Dodecanoylcarnitine [= Laurylcarnitine]), C12:1 (Dodecenoylcarnitine), C12-DC (Dodecanedioylcarnitine), C14 (Tetradecanoylcarnitine [= Myristylcarnitine]), C14:1 (Tetradecenoylcarnitine [= Myristoleylcarnitine]), C14:1-OH (Hydroxytetradecenoylcarnitine [= Hydroxymyristoleylcarnitine]), C14:2 (Tetradecadienoylcarnitine), C14:2-OH (Hydroxytetradecadienoylcarnitine, C16 (Hexadecanoylcarnitine [= Palmitoylcarnitine]), C16:1 (Hexadecenoylcarnitine [= Palmitoleylcarnitine]), C16:1-OH (Hydroxyhexadecenoylcarnitine [= Hydroxypalmitoleylcarnitine]), C16:2 (Hexadecadienoylcarnitine), C16:2-OH (Hydroxyhexadecadienoylcarnitine), C16-OH (Hydroxyhexadecanolycarnitine [= Hydroxypalmitoylcarnitine]), C18 (Octadecanoylcarnitine [= Stearylcarnitine]), C18:1 (Octadecenoylcarnitine [= Oleylcarnitine]), C18:1-OH (Hydroxyoctadecenoylcarnitine [= Hydroxyoleylcarnitine]), C18:2 (Octadecadienoylcarnitine [= Linoleylcarnitine]), PC aa (Phosphatidylcholine with diacyl residue sum), PC ae (Phosphatidylcholine with acyl-alkyl residue sum), LysoPC a (Lysophosphatidylcholine with acyl residue), SM (OH) (Hydroxysphingomyelin with acyl residue sum), SM (Sphingomyelin with acyl residue sum), Sum of hexoses (sum of Glucose, Aldohexose, L-Allopyranose, D-Allose, D-Allopyranose, D-Allose, D-Altropyranose, D-Glucopyranose, alpha-D-Glucopyranose, beta-D-Glucopyranose, D-Mannopyranose, alpha-D-Mannopyranose, L-Gulopyranose, D-Gulopyranose, D-Idopyranose, Alpha-L-Galactopyranose, alpha-D-Galactopyranose, beta-D-Galactopyranose, D-Talose, D-Talopyranose, Ketohexose, D-Psicopyranose, L-Fructofuranose, D-Fructose, D-Fructofuranose, L-Sorbopyranose, D-Sorbopyranose, D-Tagatose, D-Tagatopyranose).

**Table S2. Pearson´s correlations between components of myo-inositol metabolism (plasma myo-inositol, myo-inositol oxygenase and inositol monophosphatase 1) and plasma metabolites** (cut off at Pearson´s correlation coefficient ≥0.4)

| **Metabolite**  (µmol/l) | **Corr** | **FDR adjusted P-value** | **t-value** |  |
| --- | --- | --- | --- | --- |
| **Plasma MI** |  |  |  |  |
| Asp | 0.62 | <0.001 | 7.79 |  |
| Spermidine | 0.49 | <0.001 | 5.68 |  |
| C0 | 0.46 | <0.001 | 5.19 |  |
| Taurine | 0.41 | <0.001 | 4.42 |  |
| H1 | 0.40 | <0.001 | 4.38 |  |
| LysoPC a C161 | 0.40 | <0.001 | 4.37 |  |
| Spermine | 0.40 | <0.001 | 4.36 |  |
| **Kidney MIOX** |  |  |  |  |
| t4-OH-Pro | 0.56 | <0.001 | 6.70 |  |
| PC ae C44:5 | -0.55 | <0.001 | -6.58 |  |
| Ala | 0.55 | <0.001 | 6.50 |  |
| PC aa C34:2 | -0.54 | <0.001 | -6.37 |  |
| C16:1 | -0.54 | <0.001 | -6.33 |  |
| PC ae C36:2 | -0.53 | <0.001 | -6.27 |  |
| PC aa C32:2 | -0.53 | <0.001 | -6.23 |  |
| SM C24:1 | 0.53 | <0.001 | 6.20 |  |
| PC ae C36:1 | -0.52 | <0.001 | -6.07 |  |
| PC ae C36:4 | 0.52 | <0.001 | 6.05 |  |
| PC aa C34:3 | -0.52 | <0.001 | -6.03 |  |
| PC ae C40:6 | -0.51 | <0.001 | -5.92 |  |
| ADMA | 0.51 | <0.001 | 5.89 |  |
| PC aa C38:6 | -0.51 | <0.001 | -5.85 |  |
| PC aa C34:1 | -0.50 | <0.001 | -5.80 |  |
| Tyr | 0.50 | <0.001 | 5.70 |  |
| SM C24:0 | 0.50 | <0.001 | 5.69 |  |
| PC aa C32:1 | -0.49 | <0.001 | -5.63 |  |
| PC ae C42:4 | -0.48 | <0.001 | -5.51 |  |
| PC aa C36:4 | -0.48 | <0.001 | -5.42 |  |
| PC aa C30:0 | -0.48 | <0.001 | -5.40 |  |
| SM OH C22:2 | 0.47 | <0.001 | 5.31 |  |
| Sarcosine | 0.47 | <0.001 | 5.29 |  |
| SM OH C22:1 | 0.47 | <0.001 | 5.28 |  |
| PC aa C36:1 | -0.47 | <0.001 | -5.25 |  |
| PC aa C38:0 | -0.46 | <0.001 | -5.21 |  |
| Met-SO | 0.46 | <0.001 | 5.21 |  |
| PC aa C36:2 | -0.46 | <0.001 | -5.13 |  |
| PC aa C36:3 | -0.46 | <0.001 | -5.10 |  |
| PC ae C36:3 | -0.45 | <0.001 | -5.09 |  |
| C10:2 | -0.44 | <0.001 | -4.93 |  |
| PC aa C40:6 | -0.44 | <0.001 | -4.92 |  |
| C3 | -0.44 | <0.001 | -4.92 |  |
| PC ae C38:5 | 0.44 | <0.001 | 4.88 |  |
| PC aa C42:4 | -0.44 | <0.001 | -4.87 |  |
| PC aa C36:6 | -0.43 | <0.001 | -4.78 |  |
| PC aa C36:0 | -0.43 | <0.001 | -4.76 |  |
| Met | 0.43 | <0.001 | 4.74 |  |
| PC ae C34:3 | -0.43 | <0.001 | -4.72 |  |
| PC ae C34:1 | -0.43 | <0.001 | -4.71 |  |
| PC ae C38:6 | -0.42 | <0.001 | -4.67 |  |
| SM OH C24:1 | 0.42 | <0.001 | 4.65 |  |
| PC ae C38:4 | 0.42 | <0.001 | 4.63 |  |
| PC aa C34:4 | -0.41 | <0.001 | -4.53 |  |
| SDMA | 0.41 | <0.001 | 4.52 |  |
| LysoPC a C18:0 | 0.40 | <0.001 | 4.38 |  |
| PC ae C42:5 | -0.40 | <0.001 | -4.37 |  |
| H1 | 0.40 | <0.001 | 4.37 |  |
| PC ae C36:0 | -0.40 | <0.001 | -4.37 |  |
| PC ae C44:6 | -0.40 | <0.001 | -4.36 |  |
| PC ae C34:2 | -0.40 | <0.001 | -4.35 |  |
| t4-OH-Pro | 0.56 | <0.001 | 6.70 |  |
| PC ae C44:5 | -0.55 | <0.001 | -6.58 |  |
| Ala | 0.55 | <0.001 | 6.50 |  |
| PC aa C34:2 | -0.54 | <0.001 | -6.37 |  |
| C16:1 | -0.54 | <0.001 | -6.33 |  |
| **Muscle IMPase 1** |  |  |  |  |
| C10:1 | 0.63 | <0.001 | 8.11 |  |
| C16:1 | 0.56 | <0.001 | 6.73 |  |
| C12-DC | -0.55 | <0.001 | -6.53 |  |
| C10 | 0.53 | <0.001 | 6.23 |  |
| Dopamine | 0.51 | <0.001 | 5.86 |  |
| PC aa C38:1 | -0.47 | <0.001 | -5.31 |  |
| Spermine | 0.46 | <0.001 | 5.22 |  |
| DOPA | 0.46 | <0.001 | 5.15 |  |
| PC aa C26:0 | 0.45 | <0.001 | 4.96 |  |
| LysoPC a C14:0 | 0.43 | <0.001 | 4.82 |  |
| C3 | 0.43 | <0.001 | 4.71 |  |
| C3:1 | 0.42 | <0.001 | 4.59 |  |
| Spermidine | 0.41 | <0.001 | 4.47 |  |
| LysoPC a C18:1 | 0.40 | <0.001 | 4.42 |  |
| Muscle MI | 0.40 | <0.001 | 4.40 |  |
| C18:1-OH | 0.40 | <0.001 | 4.32 |  |

Comparison between LB and LSLS hens at all productive periods (n=100). Values are showed as Pearson´s correlation coefficients (Corr). FDR-adjusted p<0.05 as significance level was used and t-value indicates the ratio between the difference between and within both groups. Asp = aspartate; C0 = carnitine; ADMA = asymmetric dimethylarginine; Ala = alanine; C3 = Propionylcarnitine; C3:1= Propenoylcarnitine; C10:1 = Decenoylcarnitine; C10:2 = Decadienylcarnitine; C12-DC = Dodecanedioylcarnitine; C16:1 = hexadecenoylcarnitine; C18:1-OH = Hydroxyoctadecenoylcarnitine; DOPA = Dihydroxyphenylalanine; H1 = sum of hexoses; Lyso PC = lysophosphatidylcholine; Met = methionine; Met-SO = Methionine sulfoxide; MI = myo-inositol; MIOX = myo-inositol oxygenase; PC = phosphatidylcholine; SDMA = symmetric dimethylarginine; SM = sphingomyelins; t4-OH-Pro = trans-4-Hydroxyproline; Tyr = tyrosine.
